# Use of a formal consensus development technique to produce recommendations for improving the effectiveness of adult mental health multidisciplinary team meetings

**DOI:** 10.1186/s12888-015-0534-6

**Published:** 2015-07-03

**Authors:** Rosalind Raine, Caoimhe Nic a’ Bháird, Penny Xanthopoulou, Isla Wallace, David Ardron, Miriam Harris, Julie Barber, Archie Prentice, Simon Gibbs, Michael King, Jane M. Blazeby, Susan Michie, Anne Lanceley, Alex Clarke, Gill Livingston

**Affiliations:** 1 Department of Applied Health Research, University College London, London, WC1E 7HB UK; 2 Patient and Public Involvement Representative, North Trent Cancer Research Network, Consumer Research Panel, ICOSS, The University of Sheffield, Western Bank, Sheffield, S10 2TN UK; 3 Patient and Public Involvement Representative, London, UK; 4 Department of Statistical Science, University College London, Gower Street, London, WC1E 6BT UK; 5 Royal College of Pathologists, 2 Carlton House Terrace, London, SW1Y 5AF UK; 6 National Heart and Lung Institute, Imperial College London, Du Cane Road, London, W12 0HS UK; 7 Department of Cardiology, Hammersmith Hospital, Du Cane Road, London, W12 0HS UK; 8 Division of Psychiatry, University College London, Charles Bell House, 67-73 Riding House St, London, W1W 7EH UK; 9 School of Social and Community Medicine, University of Bristol, Bristol, BS8 2PS UK; 10 UCL Centre for Behaviour Change, University College London, 1-19 Torrington Place, London, WC1E 7HB UK; 11 Department of Women’s Cancer, UCL Elizabeth Garrett Anderson Institute for Women’s Health, University College London, Medical School Building, 74 Huntley Street, London, WC1E 6AU UK; 12 Department of Plastic and Reconstructive Surgery, The Royal Free Hospital, Pond Street, Hampstead, London, NW3 2QG UK; 13 Mental Health of Older People, Division of Psychiatry, University College London, Charles Bell House, 67-73 Riding House Street, London, W1W 7EH UK

**Keywords:** Multidisciplinary team, Recommendations, Chronic diseases, Consensus development method, Adult mental health

## Abstract

**Background:**

Multidisciplinary team (MDT) meetings are the core mechanism for delivering mental health care but it is unclear which models improve care quality. The aim of the study was to agree recommendations for improving the effectiveness of adult mental health MDT meetings, based on national guidance, research evidence and experiential insights from mental health and other medical specialties.

**Methods:**

We established an expert panel of 16 health care professionals, policy-makers and patient representatives. Five panellists had experience in a range of adult mental health services, five in heart failure services and six in cancer services. Panellists privately rated 68 potential recommendations on a scale of one to nine, and re-rated them after panel discussion using the RAND/UCLA Appropriateness Method to determine consensus.

**Results:**

We obtained agreement (median ≥ 7) and low variation in extent of agreement (Mean Absolute Deviation from Median of ≤1.11) for 21 recommendations. These included the explicit agreement and auditing of MDT meeting objectives, and the documentation and monitoring of treatment plan implementation.

**Conclusions:**

Formal consensus development methods that involved learning across specialities led to feasible recommendations for improved MDT meeting effectiveness in a wide range of settings. Our findings may be used by adult mental health teams to reflect on their practice and facilitate improvement. In some other contexts, the recommendations will require modification. For example, in Child and Adolescent Mental Health Services, context-specific issues such as the role of carers should be taken into account. A limitation of the comparative approach adopted was that only five members of the panel of 16 experts were mental health specialists.

**Electronic supplementary material:**

The online version of this article (doi:10.1186/s12888-015-0534-6) contains supplementary material, which is available to authorized users.

## Background

Multidisciplinary team (MDT) meetings for chronic diseases are well established in the NHS [1–4] and have been the core model for delivering mental health care for decades [5]. However, they are resource intensive, commonly occupying teams of more than a dozen professionals for several hours each week. Moreover, there is substantial diversity in their perceived purpose and their organisation, both between mental health teams and across other chronic disease MDTs [6–9]. This can partly be explained by variations in guidance provided for different conditions. Thus, cancer teams follow national guidance which sets out prescribed features of MDT meetings with respect to structure, attendance, documentation of decisions and administrative support. Cancer MDTs are nationally audited against a detailed list of indicators relating to these features [1, 10, 11]. In contrast, in mental health relatively little national guidance is available and locally determined arrangements are often advocated [3, 12]. Where recommendations are made, they tend to lack specificity. For example, guidance for Memory Clinics simply states that care plans for patients should be developed in consultation with a number of different (but unspecified) disciplines [13]. It is telling that in comparison with teams in other specialities, adult mental health teams are often idiosyncratic and produce and implement fewer MDT decisions [8].

Variations in team practice may reflect appropriate responsiveness to local needs, but may also indicate uncertainty or a lack of evidence regarding the most effective ways to conduct MDT meetings. Policy makers are now focusing on the need to improve the quality of mental health service provision through the establishment of a Mental Health Intelligence Network, based on the cancer model, to better monitor variations in provision [14]. In addition, the Care Quality Commission recently committed to developing definitions of ‘what good looks like’ in mental health services [15]. Enhancing the effectiveness of the MDT meeting, the key management decision-making body, is central to improving care overall and reducing unwarranted variations in care.

Uncertainty about determinants of MDT effectiveness is, in part, secondary to insufficient or inconclusive empirical evidence. One approach to address a lack of research or research ambiguities is to use formal consensus methods [16, 17]. These are structured facilitation techniques that measure levels of consensus among experts by synthesising their opinions [18]. In contrast to informal decision-making groups such as committees, they follow explicit steps that can be replicated. They have been used to formulate clinical practice guidelines and to inform service development in mental health [19, 20]. The three consensus methods most often used in health care research are the Delphi Method, the Nominal Group Technique, and the RAND/UCLA Appropriateness Method [16, 21, 22]. In practice, formal consensus studies often adapt components from each of these approaches to achieve their aims [17].

We applied the RAND/UCLA Appropriateness Method (RAM) to examine the extent to which it is possible to derive feasible recommendations for improving the effectiveness of mental health MDT meetings. This was part of a larger study aiming to derive recommendations which are generalisable across MDTs for patients with a range of common diseases [8]. In addition to adult mental health, we therefore also examined other disease specialties, ensuring that we included those for which comprehensive guidance exists (cancer) and others where it does not (heart failure). This allowed us to identify areas where specialities could ‘learn from one another’ and areas where condition specific recommendations were likely to be required. In this paper we present recommendations that can be implemented by adult mental health MDTs to improve their quality. Our focus is on generic recommendations for the weekly adult mental health meeting typically attended by the whole multidisciplinary team, rather than Care Plan Assessments (CPAs) and Mental Health Act assessments, which have specific requirements and formats.

## Methods

**Fig. 1:**
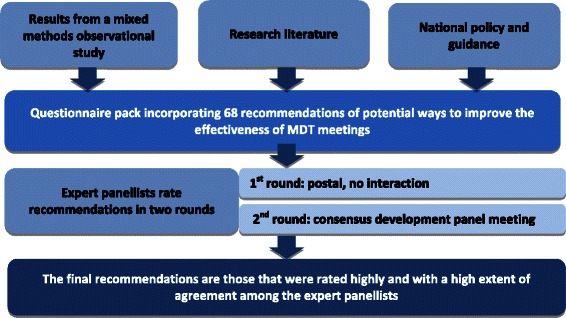
Overview of the consensus development method used

We convened a panel of experts, which included five mental health panellists with expertise in adult mental health care settings. Panellists first rated a series of recommendations on the basis of the research evidence which we provided within the context of their experience of MDT meetings and their expertise and knowledge. These ratings were done privately using a postal questionnaire. This was followed by a meeting during which panellists revised their ratings in the light of quantitative feedback on the panel’s initial ratings, a discussion of the rationale for their judgements, again within the context of their own experiences [23]. Each component of this process is described in more detail below and summarised in Fig. 1.

### Generating recommendations for the expert panel to rate

We used the following sources of evidence to generate recommendations for rating by the expert panel:

#### Research evidence

We used the results from a large mixed-methods observational study of 12 MDTs (in adult mental health, memory, cancer, and heart failure services) which we had recently undertaken [9]. In this research we examined the determinants of effective MDT decision-making and explored areas of diversity in MDT meetings. We collected data from three sources: non-participant observation of MDT meetings, semi-structured interviews with MDT professionals and patients, and clinical data from medical records. The mental health data in this study came from observations of the meetings of six Adult Community Mental Health, Early Intervention in Psychosis and Memory Clinic teams (all from the North Thames area of England, held between December 2010 and December 2012), and from semi-structured interviews with 35 mental health professionals and patients. We generated recommendations from our quantitative analysis which identified determinants of implementation of MDT decisions. We also drew on our qualitative data which explored specific practices considered effective by some teams but not used in others, appropriate issues for MDT discussions, team structure and other MDT features, and ways to improve MDT meetings and to incorporate patient preferences into MDT discussions.

In addition, we conducted a review of UK based research literature published between 1995 and May 2013 on key aspects of MDT meetings, including their purpose, structure, meeting processes, content of discussions, and the role of the patient.

#### National guidance

We identified key national policies and guidance for mental health [3, 24] and memory clinic [13, 25–28] MDTs, in addition to guidance for cancer [1, 10, 11, 29–32] and heart failure MDTs [33–35].

#### Developing recommendations

Using these data sources, we developed an initial list of recommendations of potential ways to improve the effectiveness of MDT meetings. These recommendations were refined in analytic conferences with all members of the research team to ensure that they were supported by the data. We also ensured that each recommendation was clear and precise and included only one issue for consideration.

In total we generated 68 recommendations for the expert panel to rate. These recommendations were grouped into a 16 section questionnaire, based on categories generated by a thematic analysis of the data. The final categories related to MDT purpose, structure, meeting processes, content of discussions, and the role of the patient (see Additional file 1).

#### The questionnaire

**Fig. 2:**
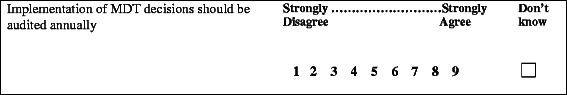
An example of a recommendation included in the questionnaire

Each of the 16 sections in the questionnaire summarised the policy, guidance and research evidence relating to each group of recommendations in that section. The full questionnaire pack is provided in Additional file 2. We instructed panellists to rate their level of support for each recommendation, according to its desirability and feasibility, by drawing on the information provided and on their own knowledge and experience of MDT meetings in their specialty [36]. Ratings were on a nine-point Likert Scale, where a rating of 1 indicated that the panellist strongly disagreed with the recommendation, a rating of 5 indicated neither agreement nor disagreement (*i.e.* depends on circumstances), a rating of 9 indicated strong agreement, and ‘don’t know’ indicated that participants did not think they were informed enough to respond. An example of a recommendation is provided in Fig. 2.

### Identification and establishment of the consensus development panel

We purposively sampled 22 health care professionals, policy-makers and patient representatives from different Trusts and regions of England, and with MDT experience of adult mental health, heart failure and cancer. Potential participants were identified by consulting the project’s Steering Group and relevant professional organisations. This approach helped to ensure that participants had credibility as experts in their field and were representative of their profession [17]. Three patient representatives (including a carer for a mental health patient) identified and recruited by our patient co-applicants, were included to bring their perspective to the discussion. Sixteen of the individuals invited agreed to participate, including five mental health experts (one psychiatrist, two psychiatric nurses, one occupational therapist and one patient representative). These panellists had expertise in an extensive range of adult mental health care settings, including adult community mental health teams, inpatient wards, day hospitals, learning disability teams, rehabilitation and recovery services, and specialist teams for substance misuse, homelessness, and forensic populations. The professional backgrounds of the other panellists are shown in Additional file 1.

#### Expert panel Round One – consensus development questionnaire

Ten weeks before the consensus development meeting, panellists were sent the first round questionnaire for completion in private.

#### Expert panel Round Two – consensus development meeting

**Fig. 3:**
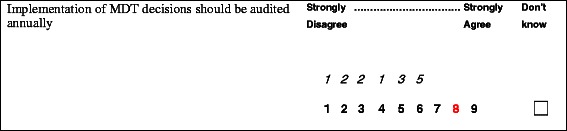
An example of a recommendation and rating from Round Two showing the distribution of Round 1 responses in italics above the Likert scale, and the respondent’s own Round 1 rating in red

The ratings from Round One were used to develop a personalised version of the questionnaire for each panel member (Fig. 3). These showed the participant’s own responses (in red) and the distribution of responses for all panellists for each item (in italics above the Likert scale).

This information was distributed to the panellists at the consensus meeting, which was chaired by the Chief Investigator who is experienced in facilitating formal consensus panels. The purpose of this meeting was to discuss those recommendations where there was a lack of consensus, to explore causes of divergent responses, and to identify where the lack of consensus was secondary to different interpretations of the recommendations. Panellists were encouraged to discuss reasons for differences in ratings before re-rating each item privately. The facilitator ensured that all participants had an opportunity to contribute during the meeting, and made clear that participants did not need to conform to the group view [16]. Where it transpired that disagreement was the result of differing interpretations, the recommendation was discussed and new wording agreed to clarify any ambiguities prior to re-rating. Where there had been consensus (defined below) in the first round ratings, recommendations were not discussed individually, although the broader discussion usually touched on the issues addressed in these recommendations and panellists were given the opportunity to comment on all recommendations before re-rating them.

With the consent of all the panellists, the meeting was audiotaped and field notes taken to ensure that we could correctly identify the profession and disease specialty associated with each discussion point made.

### Data entry and analysis

#### Data entry

Ratings were entered into a database using SPSS version 21 for Windows. There were no missing data. At each round, we double-checked 25 % of the data entered against the original paper questionnaires to ensure accuracy. No errors were detected.

#### Analysis of Round One ratings

We measured the spread of responses to each recommendation using RAND guidelines [23]. ‘Consensus’ for a panel of 16 members is defined as four or fewer panellists rating outside the 3-point region containing the median (1–3.5, 4–6.5, 7–9; *i.e.* where at least three-quarters of respondents rate in the same third of the scale). This allowed us to prioritise recommendations for discussion at the consensus meeting. 21 recommendations met this criterion and these were not discussed individually at the meeting. The remaining 47 recommendations were discussed individually.

#### Analysis of Round Two ratings: quantitative analysis

For each item, we examined the *strength* of agreement with each recommendation, and the *variation in extent* of agreement among panellists.

The *strength* of the group’s agreement with each recommendation was indicated by the median [37]. Medians between 7 and 9 indicated agreement with the recommendation, medians between 4 and 6.5 indicated uncertainty, and medians between 1 and 3.5 indicated disagreement. These ranges cover all possible medians for a 16 member panel and are based on the definitions provided in the RAM User’s Manual [23].

The group’s *variation in extent* of agreement was indicated by the mean absolute deviation from the median (MADM) [37]. This was categorised into low, moderate and high variation according to thirds of the observed MADM scores (low <1.11, moderate 1.11-1.75, and high variation >1.75).

We defined a recommendation as a final recommendation for improving the effectiveness of MDT meetings if both agreement (median between 7–9) and low variation in extent of agreement (MADM <1.11) were present.

Where recommendations were rated ‘uncertain’ (medians 4–6.5) or ‘disagree’ (medians 1–3.5), we categorised the panellists into mental health, cancer, and heart failure groups and calculated the median score for each group. This allowed us to provisionally investigate whether uncertainty or disagreement might be secondary to disease specific considerations.

#### Analysis of Round Two ratings: qualitative analysis

We transcribed the meeting in full. We conducted a thematic analysis of the meeting transcript, coding the panellists’ comments regarding each recommendation to highlight the range of views about each item discussed and to identify possible explanations for differences in ratings. We used the qualitative data to identify whether there were differences of opinion between the mental health experts and the experts from the other conditions.

### Ethical approval

The study was approved by East London Research Ethics Committee (10/H0704/68) and the National Information Governance Board for Health and Social Care (ECC 6–05 (h)/2010).

## Results

Results from the second (*i.e.* final) round of ratings are summarised and presented in Tables 1, 2, 3, 4 and 5 below. Of the 68 potential recommendations, only one needed to be re-worded for clarity before re-rating.

**Table 1:** The 21 recommendations for improving the effectiveness of mental health multidisciplinary team meetings

	Recommendation	Median	Mean absolute deviation from the median (MADM)
1	The primary objective of MDT meetings should be to agree treatment plans for patients. Other functions are important but they should not take precedence	8	0.88
2	MDT discussions should result in a documented treatment plan for each patient discussed	9	0.56
3	MDT meeting objectives should include locally (as well as nationally) determined goals	8	0.63
4	The objectives of MDT meetings should be explicitly agreed, reviewed and documented by each team	8	0.94
5	Explaining the function of the MDT meeting should be a formal part of induction for new staff	9	0.44
6	There should be a formal mechanism for discussing recruitment to trials in MDT meetings (for example, having clinical trials as an agenda item)	8	0.81
7	All Chairs should be trained in chairing skills	7	0.81
8	All new patients should be discussed even if a clear protocol exists	8.5	0.94
9	Teams should agree what information should be presented for patients discussed	9	0.56
10	All new team members should be told what information they are expected to present	9	0.38
11	The objectives of the MDT meeting should be reviewed yearly	9	1
12	Once a team has established a set of objectives, the MDT should be audited against these	7.5	0.94
13	All action points should be recorded electronically	9	0.81
14	Implementation of MDT decisions should be audited annually	8	1
15	Where an MDT meeting decision is changed, the reason for changing this should be documented	9	0.19
16	There should be a named implementer documented with each decision	9	0.38
17	Comorbidities should be routinely discussed at MDT meetings	8	0.94
18	Patients’ past medical history should routinely be available at the MDT meeting	8.5	0.56
19	The MDT should actively seek all possible treatment options, and discuss these with the patient after the meeting	9	0.44
20	Patients should be given verbal feedback about the outcome of the MDT meeting	8.5	0.94
21	Where it would be potentially inappropriate to share the content of an MDT discussion with the patient the decision not to feedback should be formally agreed and noted at the meeting	9	0.63

**Table 2:** Recommendations where strength of agreement was *agree* (median ≥7) but variation in extent of agreement was *high* (MADM score >1.75)

	Recommendation	Median	Mean absolute deviation from the median (MADM)
22	All teams should have a designated person at each MDT meeting to help identify suitable patients for clinical trials	7	1.88
23	Patients should be given feedback on all treatment options, even those rejected by the MDT	7	2.25
24	Patients should be able to choose the mode of MDT meeting feedback (*e.g.* written, phone call, in clinic)	7.5	2.19

**Table 3:** Recommendations where strength of agreement was *agree* (median ≥7) but variation in extent of agreement was *moderate* (MADM score 1.11-1.75)

	Recommendation	Median	Mean absolute deviation from the median (MADM)
25	MDT meetings should be a forum for recruiting patients to clinical trials	8	1.19
26	All MDTs should have a designated (rather than a rotating) Chair for MDT meetings	7	1.75
27	All MDTs should have a dedicated MDT coordinator/administrator	9	1.31
28	MDT Chairs should attend at least one other MDT meeting to identify approaches to improve their chairing skills	8	1.56
29	A patient list should be available for all team members to view in advance of an MDT meeting	8.5	1.31
30	Presentations should be explicitly framed in the light of a specific query or issue to be discussed	8	1.13
31	All MDTs should be audited through external peer-review	8.5	1.13
32	There should be time within MDT meetings to discuss current and emerging research and evidence only in relation to the case discussed	7.5	1.25
33	Relevant psychosocial issues for patients presented to each type of MDT should be identified and agreed by the MDT	7.5	1.44
34	The MDT member who presents the case should routinely consider psychosocial factors and ensure that relevant information is available at the meeting	8	1.19
35	Teams should be explicit about the research evidence that they are drawing on when making a decision in the MDT meeting	7	1.25
36	Patients should be given feedback on which professional groups were present when they were discussed at the MDT meeting	7.5	1.69
37	Patients should be given feedback every time they are discussed at an MDT meeting	8	1.25
38	Patients should be given written feedback about the outcome of the MDT meeting	7	1.63

**Table 4:** Medians for each disease group: recommendations rated as “uncertain” overall^a^

	Recommendation	Overall median	Mean absolute deviation from the median (MADM)	Median amongst mental health panellists N = 5	Median amongst cancer panellists N = 6	Median amongst heart failure panellists N = 5
39	The main objectives of MDT meetings should be the same across all chronic diseases	6.5	1.88	3	7	7
40	Teaching should be a function of MDT meetings provided it does not add to the length of meetings	6.5	2.31	8	6	5
41	Teaching should be a function of MDT meetings even if it means meetings will be longer	5	1.94	7	6	4
42	All treatment plans for existing patients should be agreed in an MDT meeting even if a clear protocol exists	5	2.06	7	5	2
43	Members should be allowed to not attend as long as someone from their discipline is attending and the member does not have a case to present	5	1.75	7	4.5	6
44	A list of people who are required to attend the MDT meeting should be decided locally by the team	5	2.44	6	2	7
45	A patient should only be discussed at the MDT meeting when information on comorbidity is available	4.5	2.19	2	6	6
46	A designated MDT member should speak to the patient about comorbidities before the patient is discussed at an MDT meeting	4	2.38	6	3.5	3
47	Each MDT should identify the most appropriate methods for presenting complete information on comorbidities	5	1.13	7	5	5
48	Case presentation should routinely include a brief introduction of the patient and relevant psychosocial characteristics, otherwise the case should not be discussed	6	2.38	7	4	3
49	Any MDT member who presents a case should discuss treatment preferences with the patient before the MDT meeting	5.5	2.00	7	4.5	7
50	Patient preferences regarding available treatment options should be discussed with the patient after (rather than before) the MDT meeting	5.5	1.63	5	6	8
51	Patients should not be presented at the MDT meeting unless there is someone present who has met with them at least once before the meeting, even if this postpones discussion of that patient	5	2.63	8	2	3
52	Patients should be given the opportunity to provide information in advance of the MDT meeting to ensure the information presented is accurate and comprehensive	5	2.13	7	4.5	6
53	Patients should be able to provide information by having direct access and the ability to modify their medical records	5	2.69	7	2	5
54	Patients should be given the option to provide a written summary for the meeting	5	1.88	6	3.5	3
55	Patients should be given the option to provide audio recorded input to the meeting	4.5	2.50	7	1.5	3

^a^In order to illustrate differences, numbers in green indicate agreement; and numbers in red indicate disagreement

**Table 5:** Medians for each disease group: recommendations rated “disagree” overall^a^

	Question	Overall median	Mean absolute deviation from the median (MADM)	Median amongst mental health panellists N = 5	Median amongst cancer panellists N = 6	Median amongst heart failure panellists N = 5
56	MDT meetings should be a forum for brainstorming and giving advice without necessarily reaching a decision	3	1.25	3	3	2
57	Only complex cases should be discussed in the MDT meetings (regardless of whether they are new or existing patients)	3	1.31	3	2	3
58	It is more important to discuss all patients, even if superficially, than it is to discuss a smaller number of patients in more depth	3.5	1.69	2	5	2
59	There should be time within MDT meetings to discuss current and emerging research and evidence which is not specifically related to an individual case	3.5	2.38	6	3.5	3
60	Members should be allowed to join the meeting for cases that are relevant to them and leave after the discussion of these	3	1.19	3	3	3
61	Patients’ treatment preferences should be routinely discussed at the MDT meeting and if not available the case should not be discussed	3	1.94	5	4	2
62	Patient preferences regarding available management options should be reported to the MDT meeting only if the clinician responsible for their care thinks it will alter the decision	3	2.00	5	3.5	2
63	Patients should be asked before the MDT how much they want to be involved in decision-making about their treatment	3	1.88	3	2.5	3
64	All patients should be told if they are going to be discussed at an MDT meeting before the meeting otherwise they should not be discussed	2	1.88	5	1	2
65	All patients should be explicitly given the choice of whether or not to be discussed at the MDT meeting	1.5	1.19	2	1	1
66	Patients should not be given an explicit choice, but if they express concern about being discussed at the MDT meeting they should be allowed to opt out	2	1.25	2	2	5
67	Patients should be given the option of attending MDT meetings	1	1.19	5	1	1
68	Patients should be given MDT meeting feedback only when decisions are made about their care	3	1.06	3	3	5

^a^Strength of agreement was agree for medians 7 - 9; uncertain for medians 4 - 6.5 and disagree for medians 1 - 3.5

### Recommendations where there was agreement and *low* variation in extent of agreement

We identified 21 recommendations for which there was both agreement (median ≥7) and low variation in the extent of agreement (MADM score <1.11; Table 1). This included six recommendations relating to the purpose of the meetings (Table 1, Recommendations 1–6), ten relating to meeting processes (Table 1, Recommendations 7–16), two relating to content of the discussion, and three relating to the role of the patient. Panellists from all specialties agreed that these recommendations were desirable and feasible. A printable list of these final recommendations is also provided in Box 3 of Additional file 1.

### Recommendations where there was agreement but *high or moderate* variation in the extent of agreement

There were a further 17 recommendations where there was agreement (medians between 7 and 9) but high or moderate variation in the extent of that agreement between panellists (Tables 2 and 3).

Where there were differences in opinion, the qualitative data highlighted that the main concern amongst panellists was feasibility. For example, some panellists raised concerns about the feasibility of having a designated person at each MDT meeting to identify suitable patients for clinical trials (Recommendation 22), and of giving patients feedback on all treatment options, even those rejected by the MDT (Recommendation 23): ‘*I just thought it was impractical…you would be entering into a very, very, very long conversation, creating a lot of conflict, instead of it [the feedback] being [about] the treatment you think is practical and will hopefully work*’ (doctor, mental health). Similarly, some panellists thought that allowing patients to choose the mode of MDT feedback was impractical: *‘I disagree …we can’t actually commit to sending everyone written feedback…because of time constraints’* (team manager, mental health: Recommendation 24).

Furthermore, mental health panellists did not always agree with each other. For example there were differences of opinion about whether all MDTs should have a dedicated MDT coordinator or administrator (Recommendation 27). While a mental health nurse supported this recommendation: ‘*clinicians end up doing a lot of work which could easily be done by someone else… [it would] free up their time’,* a mental health doctor argued: ‘*I’m not sure that we need them [a dedicated administrator]… the person who’s seen them needs to be able to pull all that together succinctly and report back to the team, why do you need someone to coordinate that?’*

The qualitative data also identified differences of opinion between mental health panellists and those from other specialities. For example, a mental health panellist suggested that rotating responsibility for chairing the MDT meeting allowed different members of the team to gain chairing skills: ‘*I don’t think it has to be designated, ours rotates and it works fine. I think we should learn and have those skills’* (team manager, mental health: Recommendation 26). In contrast, cancer and heart failure panellists argued that MDT meetings should have a designated Chair, or that the role should be restricted to a small number of people: ‘*it’s quite a difficult skill to chair an MDT well…the MDT becomes temporarily dysfunctional for three months whilst someone is thrown into the position of Chair who doesn’t really want to be doing it, and doesn’t necessarily have the skills to do it’* (doctor, cancer: Recommendation 26).

### Recommendations rated as ‘uncertain’

There were 17 recommendations where the strength of agreement was ‘uncertain’ (median rating was ≥4 and ≤6.5). However, calculating the median score for panellists from each discipline separately, showed that mental health panellists had rated ‘agree’ or ‘disagree’ for 13 of these (Table 4). Five of these 13 recommendations related to providing patient-centred care, for example, how information on patients’ psychosocial issues should be managed and the best ways of facilitating patient input into discussions. In contrast to cancer and heart failure panellists, mental health panellists agreed with the recommendation that patients should not be presented at an MDT meeting unless someone who has met them is present (Recommendation 51): *‘it’s different [in mental health], you can’t say anything before you’ve met the patient’* (doctor, mental health). They also agreed that patients should only be discussed when their psychosocial characteristics could be presented (Recommendation 48): *‘patients are people and I think it’s relevant as to whether their treatment is likely to impact on their social life, their quality of life, and in the other direction whether or not their context is having an impact on their treatment’* (doctor, mental health).

Similarly, mental health panellists were the only group who agreed with Recommendations 53, 54 and 55 about patients being able to provide information to the MDT by modifying their medical records and providing audio recorded input: *‘allowing them to present their viewpoints…will be further evidence of their state of mind at the time’* (patient representative, mental health).

On the other hand mental health panellists disagreed that the objectives of team meetings should be the same across all chronic diseases (Recommendation 39); *‘in a mental health MDT…people need some emotional support in managing a patient who’s quite risky. That might be very different to a more clinical orientated team who are really checking that an algorithm has been followed’* (doctor, mental health). Cancer and heart failure panellists disagreed with this, despite MDTs in different conditions being ‘*very, very different animals*’ (policy maker, cancer).

### Recommendations where there was disagreement

There were 13 recommendations which the panellists disagreed with (median <4; Table 5). A number of these centred on the role of the patient in MDT decision-making. For example panellists from all disease specialities pointed to practical and cognitive barriers to asking patients before the MDT about how much they wish to be involved in decision-making (Recommendation 63): ‘*a lot of service users have difficulties making decisions’* (team manager, mental health); ‘*I’m not sure it’s a question that many patients might be able to deal with…especially…if [they] are presenting with something that [they] don’t suspect, [they’ve] got so many things to think about’* (patient representative, cancer). Similarly, the panel disagreed with Recommendations 65 and 66 that patients should be allowed to opt out of the meeting, particularly those at risk of harm: ‘*we couldn’t do our jobs if patients could opt out. It’s not uncommon for people with mental health problems to not want to have anything to do with us!’* (doctor, mental health).

For five of the 13 potential recommendations rated as ‘disagree’ overall, the median for mental health panellists fell into the ‘uncertain’ range (Recommendations 59, 61, 62, 64, and 67).

For example, although the panel as a whole did not think that all patients should be told if they are going to be discussed in an MDT meeting (Recommendation 64) the mental health panellists were uncertain about this recommendation. In common with cancer panellists, the mental health experts were also uncertain as to whether patients should only be discussed if their treatment preferences were known (Recommendation 61): ‘*people with mental health problems don’t always have a strong preference’ (doctor, mental health).*

Finally, mental health panellists were the only group who were uncertain as to whether patients should be given the option of attending MDT meetings (Recommendation 67).

## Discussion

We demonstrate that it is possible to use formal consensus development methods to produce feasible recommendations for improving the effectiveness of MDT meetings in adult mental health services. Expert panellists from mental and physical health backgrounds agreed with 21 (31 %) of the 68 recommendations proposed and demonstrated low variation in the extent of agreement with these 21 recommendations. While previous research in this area has focused on individual conditions, our findings illustrate the value of shared learning across mental and physical health care to agree core factors for effective MDT functioning [10, 38–40]. Nonetheless, the recommendations would require modification in some contexts. For example, in Child and Adolescent services, context-specific issues such as the role of carers would need to be taken into account.

The largest category of recommendations where there was cross-specialty agreement related to MDT processes (10 of the 21 recommendations). Our findings concur with other research demonstrating the importance of clear documentation of meeting outcomes [32, 38] and regular review of meeting objectives [39]. While cancer MDTs already follow, and are audited against, national guidelines that explicitly address these issues, [7, 11] mental health MDTs do not. Our findings demonstrate agreement among experts that it would be feasible and desirable for adult mental health MDTs to also adhere to a number of these processes. Many of these recommendations require minimal additional financial resources, indicating that improvement is possible even in resource-stretched teams.

Clarity of purpose has previously been identified as a key feature of effective team working [39, 40]. However, it has been reported that mental health staff are sometimes unclear about the purpose of MDT meetings [8, 40]. We obtained consensus regarding their principal objective (*i.e.* the agreement of treatment plans) and agreement for the inclusion of both locally and nationally determined goals.

Previous research has emphasised the importance of considering patient preferences and noted the association with better MDT decision implementation rates [41–43]. However a recent survey of mental health service users by the Care Quality Commission found that only 57 % agreed that they were ‘definitely’ involved as much as they wanted to be in agreeing what care they will receive [44]. The expert panel discussed the importance of knowing patient preferences in advance of MDT meetings and of shared decision-making. Much of the discussion highlighted the complexity of these issues in terms of the most appropriate way to involve patients, and practical constraints such as the need to use involuntary treatment. The panel therefore recommended that when making a decision, the MDT should actively seek to identify all possible treatment options and discuss these with the patient *after* the meeting.

Adult mental health MDTs have certain distinctive features which explain why there was disagreement with some proposed recommendations. For example, panellists disagreed that patients should be allowed to opt out of being discussed at MDT meetings, citing concerns about the impact of this on patients at risk of harm. This may be particularly problematic if patients are unable to fully discern the adverse implications of their condition. In addition, panellists did not think that the MDT meeting should be a forum for the discussion of complex cases only.

We also identified areas where it is likely that mental health specific recommendations are required. For example, mental health panellists considered it to be imperative for someone with personal knowledge of a patient to be present when that patient is discussed by the MDT, whereas this was thought to be unnecessary in cancer and heart failure MDT meetings.

### Strengths and limitations

The novel inclusion of research evidence, policy and clinical expertise from across mental and physical health care was a key strength in this study. Highlighting the evidence and guidance available from other specialities, and bringing together diverse experts to share their different experiences, encouraged new perspectives on taken-for-granted practices and challenged assumptions regarding what can feasibly be achieved in MDT meetings. This approach allowed us to apply learning from different disease specialities to agree recommendations which might be applied generically across conditions. Our recommendations do not displace specific considerations which must be applied during CPA and Mental Health Act Assessments, *e.g.* to ensure safeguarding or to assess risk of harm to self and others. Our study design also allowed us to distinguish those issues which might be more appropriately dealt with on a condition- specific basis. A limitation of this comparative approach was that the panel of 16 experts included just five mental health specialists. The size of the panel was determined by the evidence that whilst having more group members increases the reliability of group judgement, large groups reduce the ability to elicit sufficient contributions from every member of the panel [17]. Whilst we acknowledge that five mental health panellists cannot encompass the variety of MDT meetings in mental health services, this limitation is likely to impact upon our provisional results referring to recommendations that may be better made on a condition specific basis. We therefore suggest that further examination by homogeneous (mental health) consensus development panels is needed to explicitly define those purposes and processes which are specific to mental health MDTs.

Another important strength of our study was our calculation of both the strength and extent of agreement for each recommendation. The extent (or spread) of the distribution of ratings tends to attract relatively little attention but its measurement was particularly relevant in this study because it depends on group composition [37]. Low variation in the spread of ratings was achieved in 21 recommendations which the panel agreed with. This suggests that experts from different clinical backgrounds took account of other’s opinions [45].

We chose to use the RAND/UCLA Appropriateness Method (RAM) rather than the more commonly used Delphi consensus development survey. Whilst the Delphi approach enables large sample sizes, the RAM allowed us to explore the rationale behind panellists’ ratings by qualitatively analysing the panel discussion. The level of detail provided in this face-to-face discussion enabled the production of far more informative results than would have been possible with a Delphi survey. Furthermore, to mitigate the possibility that the presentation and framing of the research evidence might influence judgements [46] we used the meeting to identify any differing interpretations of the information provided. This resulted in the rewording of just one recommendation to clarify its meaning (Recommendation 19). Finally, the panel discussion ensures that the final recommendations are concise and clear.

In determining the composition of our expert panel, we aimed to include as diverse an array of relevant ‘voices’ as possible to facilitate the exploration of comprehensive perspectives. Whilst we succeeded in including clinical, patient and policy representatives, we were unable to represent all relevant professional groups (for example, psychologists) or a wider range of patient representatives. This was partly because not all of the professionals who we approached accepted our invitation to participate, and partly because we needed to limit the number of participants included in the single panel meeting to ensure that all those present could fully participate and be heard. However an important adverse consequence was the limited patient voice. The complexity of involving patients and carers in decisions about their care was recognised by the panel and in the results relating to the role of the patient. The reliability of our results could be tested by conducting a large scale Delphi survey of a wider range of relevant ‘stakeholders’, including patients and carers [47].

Finally, audit of implemented recommendations is required to ascertain their effectiveness in practice.

## Conclusions

The availability of explicit guidance for ensuring the effectiveness of MDT meetings varies widely across mental and physical health conditions. In adult mental health care, the purpose and format of MDT meetings is largely locally formulated, while cancer teams are required to adhere to explicit, nationally determined guidance. It is tempting to justify this difference as an inevitable consequence of different disease trajectories, funding systems, patient and research contexts. Whilst it is to be expected that some disease specific guidance is necessary, our use of a formal consensus development technique enabled us to identify 21 feasible recommendations for improving the effectiveness of MDT meetings in a range of settings, including adult mental health. Comparing MDT meetings in different specialties allowed alternative ‘patterns of thinking’ to be revealed and scrutinised, prompting critical reflection on established and taken-for-granted beliefs and practices. Enhancing the effectiveness and productivity of the MDT meeting is particularly salient given the centrality of this decision-making model in the NHS and the escalating mental health burden as a proportion of all NHS activity. Thus, the application of these recommendations is important because MDT meetings are extremely resource intensive and their value to the NHS and individual patients should be maximised.

## Additional files

Additional file 1:
**Questionnaire sections, panel composition and printable version of the final 21 recommendations.**

Additional file 2:
**Round 1 Questionnaire Pack.**
