# Supplementary material for: Use of a formal consensus development technique to produce recommendations for improving the effectiveness of adult mental health multidisciplinary team meetings
Source: BMC Psychiatry. 2015 Jul 3;15:143. doi: 10.1186/s12888-015-0534-6 (PMC4489364; doi:10.1186/s12888-015-0534-6)
Supplement: Additional file 1: — Questionnaire sections, panel composition and printable version of the final 21 recommendations. [file 12888_2015_534_MOESM1_ESM.pdf]

Additional File 1

Box 1 Subsections of the questionnaire

1	The purpose of MDT meetings
2	Attendance and participation in the MDT meeting
3	Chairing the MDT meeting
4	Administrative support and the role of the MDT coordinator
5	Agreeing which patients should be discussed in MDT meetings
6	Preparing and presenting cases for discussion
7	Discussing comorbidities at MDT meetings
8	Discussing patients holistically
9	Incorporating patient preferences about treatment into MDT discussions
10	Patient awareness of MDT meetings
11	Patient attendance at MDT meetings
12	Providing feedback to patients on the outcome of MDT discussions
13	The role of research and evidence in MDT meetings
14	Teaching as a function of MDT meetings
15	Recruitment to trials in MDT meetings
16	Monitoring the quality of MDT meetings

Box 2 Professional backgrounds of panellists

Profession	Area of expertise
Nurse	Adult Mental Health
Nurse	Adult Mental Health
Occupational Therapist	Adult Mental Health
Doctor	Adult Mental Health
Carer representative	Adult Mental Health
Nurse	Heart Failure
Nurse	Heart Failure
Doctor	Heart Failure
Doctor	Heart Failure
Patient representative	Heart Failure
Nurse	Cancer
Nurse/Policy	Cancer
Doctor/Policy	Cancer
Doctor	Cancer
Doctor	Cancer
Patient representative	Cancer

Box 3. Recommendations to improve the effectiveness of multidisciplinary team meetings for patients with chronic diseases (printable version)

The purpose of MDT meetings

1. The primary objective of MDT meetings should be to agree treatment plans for patients. Other functions are important but they should not take precedence.
2. MDT discussions should result in a documented treatment plan for each patient discussed.
3. MDT meeting objectives should include locally (as well as nationally) determined goals.
4. The objectives of MDT meetings should be explicitly agreed, reviewed and documented by each team.
5. Explaining the function of the MDT meeting should be a formal part of induction for new staff.
6. There should be a formal mechanism for discussing recruitment to trials in MDT meetings (for example, having clinical trials as an agenda item).

MDT meeting processes

7. All new patients should be discussed in an MDT meeting even if a clear protocol exists.
8. All Chairs should be trained in chairing skills.
9. Teams should agree what information should be presented for patients brought for discussion in an MDT meeting.
10. All new team members should be told what information they are expected to present on patients they bring for discussion in an MDT meeting.
11. The objectives of the MDT meeting should be reviewed yearly.
12. Once a team has established a set of objectives for the meeting, the MDT should be audited against these goals (e.g. biannually).
13. All action points should be recorded electronically.
14. Implementation of MDT decisions should be audited annually.
15. Where an MDT meeting decision is changed, the reason for changing this should always be documented.
16. There should be a named implementer documented with each decision.

Content of discussion in MDT meetings

17. Comorbidities should be routinely discussed at MDT meetings.
18. Patients' past medical history should routinely be available at the MDT meeting.

The role of the patient in MDT meetings

19. The MDT should actively seek all possible treatment options, and discuss these with the patient after the meeting.
20. Patients should be given verbal feedback about the outcome of the MDT meeting.
21. Where it would be potentially inappropriate to share the content of an MDT discussion with the patient (e.g. where it may lead to unnecessary anxiety or disengagement from services), the decision not to feedback should be formally agreed and noted at the meeting by the team.
