# Supplementary material for: Use of a formal consensus development technique to produce recommendations for improving the effectiveness of adult mental health multidisciplinary team meetings
Source: BMC Psychiatry. 2015 Jul 3;15:143. doi: 10.1186/s12888-015-0534-6 (PMC4489364; doi:10.1186/s12888-015-0534-6)
Supplement: Additional file 2: — Round 1 Questionnaire Pack. [file 12888_2015_534_MOESM2_ESM.pdf]

Additional File 2: Round 1 Questionnaire Pack

UCL DEPARTMENT OF APPLIED HEALTH RESEARCH

Improving the effectiveness of multidisciplinary team meetings for patients with chronic diseases

Consensus Development Group Meeting

[dateand location of meeting]

Please return in the SAE by [date] to:

[Researcher name]
Department of Applied Health Research
University College London,
1-19 Torrington Place
London WC1E 7HB

[Insert address]

Dear [insert name]

Re: Improving the effectiveness of multidisciplinary team (MDT) meetings for patients with chronic diseases (NIHR funded research)

Many thanks for agreeing to take part in our Consensus Development Panel on improving the effectiveness of MDT meetings.

As you know, your participation in this study involves two stages:

1. Responding to the questions in the enclosed booklet.
2. Taking part in a Consensus Development Meeting on the 10th of September 2013, 11.30am-3pm.

Instructions and further information about each stage are detailed below.

1. Completing the questions in the booklet

The booklet briefly outlines possible areas in which MDTs could be modified to improve their effectiveness. However, as described in the booklet, the research evidence and guidance is insufficient to allow the generation of recommendations. We have therefore invited you and your fellow panellists to draw on your knowledge and experience, in addition to the information provided, to give your views about each area.

We would like you to:

- Rate your personal level of support for each of the recommendations provided on a scale of 1 to 9, where:

  1=strongly disagree
  5=neither agree nor disagree, i.e. depends on circumstances
  9=strongly agree
  Don't know = you do not think you are informed enough to answer the question.

- Please think about what would be feasible rather than merely desirable recommendations.

- **There are no right or wrong answers.** We have recruited a range of professional groups and experts to capture diverse viewpoints.
- There are also some `open questions`. Please write brief responses. Where possible, we will then convert your responses into recommendations for consideration at the Consensus Development Meeting on 10 September.

This process will take about 90 minutes. All the questions do not need to be answered in one sitting.

**Please return the questionnaire in the enclosed pre-paid envelope by Friday the 19th of July**.<br>This will allow us to collate all replies in time for the consensus development meeting.

2. The Consensus Development Meeting

This will take place on [date and location] (map and travel instructions enclosed).

At the Consensus Development Meeting, we will remind you of your previous rating for each question, and also provide you with the distribution of ratings from other panellists for each recommendation. The feedback will be personalised so that each participant receives their own first round ratings, but the personalised rating will not be revealed to other panellists.

During the meeting we will discuss each of the recommendations, focusing on those where there is little consensus among the participants. When differences in first round questionnaire ratings have resulted from ambiguity in the wording of the question, we will agree a revised wording before making a second round rating. This will provide everyone with an opportunity to review their initial ratings in the light of the points raised and rate the recommendations again. There is no obligation to alter your initial rating.

The meeting will be audiotaped, provided all participants consent to this, to allow us to capture the particular components of the discussion that explain continuing lack of consensus.

Please call or email if you would like to discuss any aspect of the task, or if you need further information.

We look forward to meeting you at the Consensus Development Meeting.

Best wishes,

[Chief investigator]

Contents

Summary of Data Sources 5

1 Theme: The purpose of MDT meetings 6

2 Theme: Attendance and participation in the MDT meeting 10

3 Theme: Chairing the MDT meeting 13

4 Theme: Administrative support and the role of the MDT coordinator 15

5 Theme: Agreeing which patients should be discussed in MDT meetings 17

6 Theme: Preparing and presenting cases for discussion 20

7 Theme: Discussing comorbidities at MDT meetings 24

8 Theme: Discussing patients holistically 27

9 Theme: Incorporating patient preferences about treatment into MDT discussions 29

10 Theme: Patient awareness of MDT meetings 31

11 Theme: Patient attendance at MDT meetings 34

12 Theme: Providing feedback to patients on the outcome of MDT discussions 37

13 Theme: The role of research and evidence in MDT meetings 40

14 Theme: Teaching as a function of MDT meetings 42

15 Theme: Recruitment to trials in MDT meetings 43

16 Theme: Monitoring the quality of MDT meetings 46

REFERENCES 47

Summary of Data Sources

The recommendations you are rating are based on the following data sources:

1. Data collected as part of the NIHR funded study "**Improving the effectiveness of MDT meetings for patients with chronic diseases**". This study collected:

   i) Quantitative data, including:
   - Data on 3184 patients discussed at the MDT meetings of 12 chronic disease MDTs across North London and Essex. These comprised 4 **Cancer** teams (gynaecological, skin and 2 haematological), 4 **Mental Health** teams, 2 **Memory Clinic** teams and 2 **Heart Failure** teams. Data were collected between December 2010 and May 2012.
   - Data on patient clinical and socio-demographic characteristics, MDT discussions, decision implementation, and the 'Team Climate Inventory'. This is a validated survey of the perceived atmosphere or 'climate' of a team, which was completed by members of each team.

   ii) Qualitative data, including:
   - non-participant observation of 370 MDT meetings (of the 12 teams)
   - 53 interviews with MDT members across the 4 specialties (Cancer, Memory Clinic, Heart Failure and Mental Health)
   - 20 interviews with patients across the 4 specialties
   - the responses of MDT team members to a survey question about what they would change about their MDT meeting.

2. The research literature on MDTs with a focus on the areas highlighted in our study as requiring further investigation.

3. UK policy and guidance on these areas.

Please note that for the sake of brevity we use the term `patient` to include both patients (in Cancer and Heart Failure) and service users (in Mental Health).

Theme: The purpose of MDT meetings

1.1 Key points from policy and guidance

Cancer policy states that the prime function of an MDT meeting is clinical, and is to ensure that all the relevant information about the patient is made available, all the relevant treatment options are considered, and decisions about patient care are documented. (1)

Mental Health policy states that assessments and reviews should be routinely discussed in a weekly meeting where actions are agreed and changes in treatment discussed by the whole team, providing peer review and support. (2)

There is no policy outlining the purpose of MDT meetings in **Memory Services**, however, policy does state that there should be opportunities for the multidisciplinary team to meet at least once a week to discuss clinical matters. (3)

Commissioning guidelines for **Heart Failure** services recommend that regular MDT discussions include review of outcomes, current care plans and possible improvements and potential discharge or referrals. (4)

1.2 Key points from the research literature

Research has highlighted the importance of clarity of purpose for effective team working and care of patients. (5)

A recent study of Mental Health teams found that the purpose of meetings was implicit rather than explicit and that there was rarely a written agenda. (5)

1.3 Our research findings

Quantitative findings

Two subscales of the Team Climate Inventory are relevant to a team's clarity of purpose: Team Vision (extent which members believe the team has a clear, shared, attainable vision) and Task Orientation (extent to which members believe they engage in reflective practice and constructive controversy).

Memory Clinics scored highest on both of these constructs, followed by **Cancer**, **Heart Failure** and finally **Mental Health** teams.

There was wide variation across specialties as to whether MDT discussions resulted in a treatment decision. Decisions were most likely to be made in **Memory Clinics** (90% of discussions) followed by **Heart Failure** (85%), **Cancer** (77%) and **Mental Health** (42%). Where no decision was made, this was most commonly the result of insufficient information (Cancer) or because the output was feedback to the team to ensure continuity of care without seeking to resolve a specific query (Mental Health).

Qualitative findings

Meetings served a variety of functions including decision-making, data collection, peer support, sharing information, sharing responsibility, gathering advice, "brainstorming", education, reflecting on practice, discussion of service issues, and team management.

Several Mental Health MDT members highlighted a need for clarification and review of the purpose of MDT meetings.

When asked about the primary function of the team meeting, member interviewees provided a range of answers presented in the table below.

Cancer	Mental Health	Memory	Heart Failure
• agreeing treatment plans<br>• providing holistic multidisciplinary review<br>• meeting audit requirements<br>• diagnosis<br>• checking trial eligibility	• information sharing<br>• seeking advice<br>• decision making<br>• liaising with peers<br>• feedback on on-going work to ensure team awareness for cover and shared responsibility<br>• ensuring quality & consistency<br>• raising concerns & providing support	• agreeing treatment plans<br>• diagnosis<br>• liaising with peers<br>• seeking advice<br>• team cohesiveness<br>• validation of earlier decisions	• ensuring quality<br>• improving care through consensus<br>• information gathering<br>• coordinating different specialties

Table 1. The primary purpose of the meetings (MDT members' responses)

Degree to which focus is multidisciplinary

In some teams, members said that their meetings were medically dominated and that it was not a truly multidisciplinary meeting.

Several non-medical MDT members commented that they did not feel a sense of ownership at the meeting and did not always feel able to contribute freely about patient management or to contribute to the design of the meeting.

Several MDT members said that they would like further instruction and clarity around what was expected of them in team meetings.

Balancing decision making and other functions

There was variation in the degree to which the MDT meeting was focused on formulating treatment decisions. This is reflected in the variation across MDTs in the number of discussions that did not result in a decision (see Section 1.3).

In the **Cancer** and **Memory** MDT meetings a higher proportion of new cases received decisions according to standard protocols compared to those discussed in **Mental Health** MDT meetings. The latter were more focused on established clients, raising case-specific complexities and social challenges. They made little reference to established guidance. These discussions of broad social issues contrasted with the more biomedical focus of **Cancer** and **Heart Failure** MDT meetings.

In many teams, weekly MDT meetings were one of a number of discussion forums including daily handover meetings and weekly business meetings. Clinical decision-making was distributed across these meetings in different ways and the MDT was not considered the main forum for decision-making in all teams.

Members of **Heart Failure**, **Memory Clinics** and **Haematological Cancer** MDTs reported that they would like to use the meeting for more feedback regarding the outcomes of earlier decisions.

Some members of **Mental Health** and **Memory** MDTs reported that they would like more time for emotional support around complex cases.

There was some concern that overly prescriptive protocols and procedures could be restrictive, reducing the ability to be appropriately responsive to changing circumstances.

1.4 Questions

The main objectives of MDT meetings should be the same across all chronic diseases	**Strongly Disagree** ....**Strongly Agree**<br>1 2 3 4 5 6 7 8 9	**Don't know**<br>☐

MDT meeting objectives should include locally (as well as nationally) determined goals	**Strongly Disagree** .... **Strongly Agree**<br>1 2 3 4 5 6 7 8 9	**Don't know**<br>☐

The primary objective of MDT meetings should be to agree treatment plans for patients. Other functions are important but they should not take precedence.	**Strongly Disagree** ....**Strongly Agree**<br>1 2 3 4 5 6 7 8 9	**Don't know**<br>☐

MDT meetings should be a forum for brainstorming and giving advice without necessarily reaching a decision	**Strongly Disagree ....Strongly Agree**<br>1 2 3 4 5 6 7 8 9	**Don't know** ☐
The objectives of MDT meetings should be explicitly agreed, reviewed and documented by each team	**Strongly Disagree ....Strongly Agree**<br>1 2 3 4 5 6 7 8 9	**Don't know** ☐
Explaining the function of the MDT meeting should be a formal part of induction for new staff	**Strongly Disagree ....Strongly Agree**<br>1 2 3 4 5 6 7 8 9	**Don't know** ☐
MDT discussions should result in a documented treatment plan for each patient discussed	**Strongly Disagree ....Strongly Agree**<br>1 2 3 4 5 6 7 8 9	**Don't know** ☐

1 Theme: Attendance and participation in the MDT meeting

2.1 Key points from policy and guidance

There is a list of professionals who should be part of the ‘core team’ for each **Cancer** specialty. This includes oncologists, clinical nurse specialists, pathologists, imaging specialists and where applicable, surgeons and/or dermatologists. There may be additional core members agreed locally by the team. (6)

Mental Health policy states that a CMHT requires the skills of nursing, social work, psychology and medicine, and that weekly team meetings should include the whole team, including the consultant psychiatrist. The policy acknowledges that flexibility may be necessary, given different team structures which require different staffing structures (2).

Guidance for **Memory Clinics** states that care plans for patients should be developed in consultation with a number of different (unspecified) disciplines. (7)

There are no specific guidelines for who should attend **Heart Failure** MDT meetings.

2.2 Key points from the research literature

Maximising the collective knowledge and skills of team members improves care outcomes. (8)

However, not all members participate equally in MDT discussions. (9)

Research has found that barriers to effective team working include issues of status and the diverse objectives of different professional groups (e.g. doctors and nurses). (10)

2.3 Our research findings

Quantitative findings

Mean numbers of core members attending their MDT meeting ranged from 5 to 28. There were between one and seven different professional categories represented at each meeting (with a median of 4 professional categories).

The majority (78%) of members surveyed agreed that the MDT meeting was an effective use of their time.

However, our quantitative analysis found a decreased likelihood of decision implementation was associated with an increasing number of professional categories present at the meeting. This trend was not consistent across teams and was mostly accounted for by the **Memory** and **Mental Health** MDT meetings.

Qualitative findings

MDT meetings for **Memory** and **Mental Health** were usually attended by the whole team, and characterised by discussions in which most members participated

MDT meetings for **Cancer,** and to some extent **Heart Failure**, often included additional people who were not part of the core team. This included individuals from other clinical teams who had input into a specific patient’s care and others who were observing, for example, visiting members of staff, students or junior doctors. Most core members

participated in discussions, but there was a tendency for discussion to be medically dominated.

Some MDT members interviewed suggested widening the representation in MDT meetings, e.g. including a representative from Primary Care, and from other professional groups to balance medical dominance in the team.

It was more common in **Cancer** meetings for some members to attend only specific parts of the meeting.

We found that in all teams there were issues with core members having to miss meetings due to other work commitments.

2.3.1 Arguments in favour of all MDT members attending the entire MDT meeting every week

MDT members value the MDT meetings for bringing together different sources of professional expertise, experience, different cultural perspectives, and knowledge of specific patients.

There are longer term benefits of involving everyone in the meetings, including improved team work and communication and peer education.

It can be difficult for members of a particular discipline to challenge the dominant group if they are outnumbered.

2.3.2 Arguments against all MDT members attending the entire MDT meeting every week

Increased participation does not guarantee new and relevant information. In combination with less directive chairing, it may lead to a lack of focus and a reduced tendency to make clear decisions.

Not all members who attend the meeting contribute to discussions. In practice, some MDT meetings are heavily reliant on a few key individuals and there is limited input from other professions.

In **Mental Health** MDTs in particular, where nurses, occupational therapists and social workers all adopt "care-coordinator" roles, many reported that they tend to contribute in a generic way rather than providing specialist input.

MDT meetings are time and resource intensive – having more professional contributions may slow down the meeting.

Some MDT members said that those who do not contribute to specific cases are wasting their time by being at the meeting when they could be seeing patients.

2.4 Questions

Members should be allowed to join the meeting for cases that are relevant to them and leave after the discussion of these	**Strongly Disagree ....Strongly Agree**	**Don't know** ☐

	1 2 3 4 5 6 7 8 9	

Members should be allowed to not attend as long as someone from their discipline is attending and the member does not have a case to present	**Strongly Disagree ....Strongly Agree**<br>1 2 3 4 5 6 7 8 9	**Don't know**

A list of people who are required to attend the MDT meeting should be decided locally by the team	**Strongly Disagree ....Strongly Agree**<br>1 2 3 4 5 6 7 8 9	**Don't know**

2 Theme: Chairing the MDT meeting

3.1 Key points from policy and guidance

Guidance on effective MDT working in **Cancer** states that there should be an MDT Chair who is responsible for the organisation and running of the MDT meetings, and that there should be access to training in chairing skills. (11)

There is no specific guidance in **Mental Health, Memory** or **Heart Failure** on how MDT meetings should be chaired.

3.2. Key points from the research literature

In a survey of 2054 cancer MDT members, the majority (81%) agreed that 'the same individual should chair the meeting on a regular basis'. (12)

3.3. Our research findings

Qualitative

In some teams a designated person chaired every meeting. In others, the Chair rotated between team members on a weekly basis. Other teams did not have a pre-defined chairing system and different senior members took the lead on different occasions (sometimes changing during a meeting).

Where teams had a designated Chair, this was either a Consultant or the Team Manager.

3.3.1 Arguments in favour of having a designated (rather than a rotating) Chair

In teams with a rotating Chair there were several occasions when members did not know who was due to chair specific meetings. This caused delays.

Some MDT members said that having a rotating Chair is problematic as some team members lack the authority or training to keep presentations succinct and discussions focused.

3.3.2 Arguments against having a designated (rather than a rotating) Chair

It is a significant responsibility for one member of the team to Chair the meeting every week. It was suggested that a rotating Chair would help to reduce this burden.

Rotating the Chair gives more people the chance to acquire chairing skills.

3.4 Questions

All MDTs should have a designated (rather than a rotating) Chair for MDT meetings	**Strongly Disagree** ....**Strongly Agree**<br><br>1 2 3 4 5 6 7 8 9	**Don't know**<br><br>☐

All Chairs should be trained in chairing skills	**Strongly Disagree ....Strongly Agree**<br>1 2 3 4 5 6 7 8 9	**Don't know**<br>☐

MDT Chairs should attend at least one other MDT meeting to identify approaches to improve their chairing skills	**Strongly Disagree ....Strongly Agree**<br>1 2 3 4 5 6 7 8 9	**Don't know**<br>☐

Theme: Administrative support and the role of the MDT coordinator

4.1 Key points from policy and guidance

In **Cancer**, MDT coordinators are classified as core members of MDTs and perform a range of administrative duties relating to the planning and facilitation of the MDT meeting. Their specific responsibilities vary by team and they may work with more than one team depending on workload. (13)

Mental Health policy provides recommendations regarding the amount of dedicated administrative support required based on a given caseload size and complexity. (2) However, the specific duties of this role are not specified.

Similarly, the **Memory Services** National Accreditation Programme Standards dictate that a memory service MDT should have access to "adequate administrative support" without specifying what this would involve. (3)

There is no specific guidance on MDT meeting administration in **Heart Failure** teams.

4.2 Key points from the research literature

Previous research has acknowledged the importance of the MDT coordinator role in **Cancer** and described it as essential to MDT decision making. (14)

95% of cancer MDT members surveyed reported having MDT coordinators of whom 49% worked with more than one team. (12)

A survey of breast cancer teams in England found that most units believed MDT coordinators were essential to organising and running the MDT meeting. (15)

4.3 Our research findings

Qualitative findings

There is wide variation in how MDT meetings are organised in different specialties. In keeping with policy, all **Cancer** teams studied had dedicated MDT coordinators, while in **Heart Failure**, **Memory** and **Mental Health**, administrative duties were undertaken by managers, healthcare professionals and administrators.

Cancer MDT coordinator responsibilities included distributing lists of patients for discussion, operating IT equipment for imaging and video-links, recording attendance, ensuring medical records and test results are available at the meeting, and monitoring patient review periods and compliance with waiting time targets. Some MDT coordinators also recorded the outcome of each MDT discussion.

In teams without MDT coordinators, administrative support was more limited and MDT related administration was largely undertaken by the professionals themselves in various ways. For example, documentation of MDT decisions varied greatly, ranging

from doctors writing discussion summaries in medical records, to managers handwriting minutes in a notebook.

4.3.1 Arguments in favour of having dedicated MDT coordinators to support meetings

Individuals from MDTs with coordinators reported that they were 'indispensable'.

In three of the teams without coordinators, there was evidence of inadequate communication about the meeting, with team members attending for meetings that had been cancelled.

There was evidence that having an MDT coordinator increased the efficiency of the meetings. In teams without coordinators, disruption and delays occurred due to people having to leave to get medical records, test results and other documents.

MDT coordinators usually collate patient lists and keep track of how frequently patients are discussed. In teams without coordinators, there was sometimes confusion as to whether a patient had been discussed at a previous meeting, resulting in delays.

There was evidence that having an MDT coordinator improved record keeping, allowing for better monitoring of action points and facilitating audit.

Some professionals said they did not wish to perform administrative tasks such as taking minutes at meetings because it prevented them from participating fully in discussions.

Having a coordinator could increase clarity around different roles. One manager reported confusion regarding whether he was in an administrative or leadership position.

The presence of a coordinator allows the Chair and other MDT members to focus on their appropriate roles.

4.3.2 Arguments against having dedicated MDT coordinators to support meetings

Resource constraints preclude the ability to recruit a coordinator in some MDTs.

There may be scope for improving administration within teams without necessarily recruiting additional staff. For example, by introducing systems for record keeping and data collection and building responsibilities (e.g. for bringing relevant documents to each meeting) into job plans.

Having an MDT coordinator doesn't necessarily eliminate administrative and procedural problems (e.g. problems with IT equipment, and discussing patients before sufficient information is available).

An important part of the **Cancer** MDT coordinator role is ensuring that pathology and radiology teams are aware, in advance, of all patients whose histological specimens and radiological images are to be reported before the meeting, and ensuring the results are available for presentation. This information is not routinely required in **Mental Health** MDTs so there may be less need for dedicated administrative support.

4.4 Question

All MDTs should have a dedicated MDT coordinator/administrator	**Strongly Disagree** ....**Strongly Agree**<br>1 2 3 4 5 6 7 8 9	**Don't know**<br>☐

Theme: Agreeing which patients should be discussed in MDT meetings

5.1 Key points from policy and guidance

Cancer policy states that all **new** cancer cases should be reviewed by an MDT, and that a local policy should specify at what other stages in the patient pathway patients are referred back for discussion. (6, 16, 17)

Guidance from the Academy of Royal Colleges states that because there is only a finite amount of time in **Cancer** MDT meetings, patients should be triaged and more time devoted to more complex cases, for example prioritising patients with psychosocial issues or comorbidities which may affect their management. (1)

Mental Health policy states that service user assessments and reviews should be routinely discussed by the whole team in a systematic manner, and that the team should not be reliant on individual members flagging up problems. (2) However, it is also acknowledged that not all decisions will require consultation with the wider team. (18)

There is no specific guidance on which patients should be discussed in **Memory** or **Heart Failure** MDTs.

5.2 Key points from the research literature

Strategies to reduce the number of patients discussed in MDT meetings may allow more time for complex cases that require extensive discussion. However, the suggestion that discussion of fewer cases would lead to better MDT working may be overly simplistic. An observational study of Cancer MDT meetings found that a higher number of cases discussed was associated with better overall team performance. (19) This was attributed to better case preparation and discussion.

5.3 Our research findings

Quantitative findings

MDTs discussed an average of between 4 and 49 patients at weekly MDT meetings, which ran for between 1-3 hours.

The mean number of patients discussed by each team did not necessarily relate to the length of the meeting, for example a team discussing an average of 4 patients per week had the same length of meeting as a team discussing 22 patients per week.

Qualitative findings

The teams adopted different approaches to selecting which patients to discuss at each meeting. These included:

- running through the whole team caseload at each meeting
- routinely discussing all new cases, as well as complex cases or those identified in advance by clinicians with specific concerns
- relying on individual team members to raise patients if, or as and when, they thought it was necessary
- discussing only specific groups of patients, for example all inpatients.

There were mixed views from MDT members regarding which patients should be discussed at the meeting, with some suggesting that all cases should be discussed and others that only complex cases should be discussed. It was also suggested that more time could be spent on complex cases if not all cases were discussed in detail.

5.3.1 Arguments in favour of discussing all patients in an MDT meeting

Patients who are not discussed in an MDT meeting might miss out on the benefits of a multidisciplinary discussion, including consideration of different options and a decision making process that is transparent and subject to alternative viewpoints.

It is not always possible to determine in advance which patients will benefit from an MDT discussion.

The MDT meeting is an opportunity to liaise with several colleagues at once, thus saving time.

There is scope to prioritise patients to allow for more time for complex patients, without excluding other patients from the MDT meeting completely.

5.3.2 Arguments against discussing all patients in an MDT meeting

Time constraints mean that there is not always time to discuss all patients in sufficient detail.

Often the same decision would be made regardless of an MDT discussion, for example where protocols exist.

Not all patients want to be discussed in an MDT meeting, because they do not like the thought of being discussed by people they have never met.

5.4 Questions

All **new** patients should be discussed in an MDT meeting even if a clear protocol exists	**Strongly Disagree ....Strongly Agree**<br>1 2 3 4 5 6 7 8 9	**Don't know**<br>☐

Only complex cases should be discussed in the MDT meetings (regardless of whether they are new or existing patients)	**Strongly Disagree ....Strongly Agree**	**Don't know**<br>☐

	1 2 3 4 5 6 7 8 9

If yes,

Do you have any suggestions for criteria for identifying 'complex cases' so that they can be identified before the meeting?

	Strongly Disagree ....Strongly Agree	Don't know
All treatment plans for existing patients should be agreed in an MDT meeting even if a clear protocol exists	1 2 3 4 5 6 7 8 9	☐

	Strongly Disagree ....Strongly Agree	Don't know
It is more important to discuss all patients, even if superficially, than it is to discuss a smaller number of patients in more depth	1 2 3 4 5 6 7 8 9	☐

Theme: Preparing and presenting cases for discussion

6.1 Key points from policy and guidance

Guidance states that an effective **Cancer** MDT is one in which:

- it is clear who wants to discuss a particular patient and why they are being discussed (11)
- members know what information they will be expected to present on each patient so that they can prepare, and if necessary, share this information at the meeting (11)
- a list of patients to be discussed is made available in advance of the meeting, to give adequate time to provide a robust radiological opinion (20)
- a locally agreed set of information is presented on each patient including diagnostic information (pathology and radiology), clinical information (including co-morbidities, psychosocial and specialist palliative care needs) and patient history, views and preferences. (11)

Detailed guidance on the structure and operation of MDT meetings does not exist for **Heart Failure**, **Memory** or **Mental Health** MDTs.

6.2 Key points from the research literature

A national survey of cancer MDT members found that over 90% thought that an agenda and patient list should be circulated prior to an MDT meeting. (12)

Improving the process of registering patients for an MDT meeting, and organising and tracking test results and investigations in advance of the meeting, can reduce delays in treatment planning. (21)

Previous research has assessed the quality of information presentation in cancer MDT meetings. This found that the majority of the time for each case was taken up with information presentation, and that the best quality information related to case histories and radiology. In contrast, information about patients' views and comorbidities/psychosocial issues was provided in the least amount of detail. (22)

6.3 Our research findings

Qualitative findings

Some MDTs had a formal mechanism for adding patients to a patient list. This list was circulated to members in advance of the meeting, either by an MDT coordinator or by a clinical member of the team. Other teams had an ad hoc approach which relied on individual team members bringing cases for discussion to the MDT meeting as and when they thought it necessary.

When patients were presented in the MDT meeting, a relatively structured approach was used in the **Cancer** and **Heart Failure** teams. This involved a brief case presentation of predominantly clinical information, followed by review of any relevant imaging or pathology results, and discussion and/or decision-making. Some teams allocated space on the patient list to record the specific reason for bringing each patient for discussion. This contrasts with the approach taken by **Mental Health** and **Memory** teams, where case presentations were less structured but generally more holistic, and focused on the patient and their personal and social circumstances as well as (and at times instead of) biomedical information.

Some MDTs were characterised by lengthy presentations in which information was not prioritised according to its relevance for decision making. In some cases, MDT members were unclear as to why a patient had been presented because no queries were raised.

The need for more focus in presentations was raised by MDT members from all types of team (**Cancer**, **Mental Health**, **Memory** and **Heart Failure**).

6.3.1 Arguments in favour of agreeing a list of patients to be discussed in advance of an MDT meeting

Circulating a patient list in advance gives the opportunity to collate all the necessary material needed to discuss a patient.

A patient list ensures that no patients are forgotten about.

It also enables the Chair to better manage the meeting, because s/he knows how many patients need to be discussed and can prioritise cases appropriately.

It can encourage MDT members to define a specific query in advance of the meeting, leading to improved focus during the presentation.

Having an agreed patient list does not necessarily mean there is no scope for flexibility. In some teams there was the opportunity at the end of the meeting to raise any patients who were not on the list circulated before the meeting.

6.3.2 Arguments against agreeing a list of patients to be discussed in advance of an MDT meeting

It is resource intensive, because it relies on one member of the team collating and coordinating this every week.

An informal approach where all team members are asked in the meeting if there is anyone they want to discuss can be more inclusive, creating the expectation that all individuals should participate in the discussions.

Sticking too rigidly to a list can disadvantage some groups of patients, for example the order patients are put on the list is likely to impact on the length of time they get discussed.

For MDTs which do not routinely review radiology or pathology or other diagnostic information in the MDT, there may be less need to prepare cases for discussion in advance of the meeting.

The need to compile a list can result in patients being placed on the list before all the information is available. This can lead to time wasting during the MDT meeting.

6.3.3 Arguments in favour of having an agreed format for structuring patient presentations

It leads to more efficient use of time in the meeting by highlighting salient information and avoiding unnecessary detail.

A well-structured presentation can open up a discussion to all team members, because everyone has enough information to comment constructively on the case.

Presenting only the salient points of a case keeps the meeting focused and more interesting.

An agreed format helps those team members who are unsure about what information they should present at the MDT, particularly for complex patients.

Some members expressed frustration about long presentations, which failed to prioritise relevant information.

An agreed format for presentations could be used to prompt presenters to consider non-clinical information, for example patients' social circumstances and patient perspectives about their management.

6.3.4 Barriers to having an agreed format for structuring patient presentations

Time constraints are a real issue in some meetings, and not all cases necessarily need a full presentation or discussion.

Missing information is a common problem for some teams, so it may not always be possible to present according to an agreed format.

Different members of the team prioritise different types of information as important.

6.4 Questions

	Strongly Disagree ....Strongly Agree	Don't know
A patient list should be available for all team members to view in advance of an MDT meeting	1 2 3 4 5 6 7 8 9	☐

	Strongly Disagree ....Strongly Agree	Don't know
Teams should agree what information should be presented for patients brought for discussion in an MDT meeting	1 2 3 4 5 6 7 8 9	☐

All new team members should be told what information they are expected to present on patients they bring for discussion in an MDT meeting	**Strongly Disagree** ....**Strongly Agree**<br><br>1 2 3 4 5 6 7 8 9	**Don't know**<br><br>☐

Presentations should be explicitly framed in the light of a specific query or issue to be discussed	**Strongly Disagree** ....**Strongly Agree**<br><br>1 2 3 4 5 6 7 8 9	**Don't know**<br><br>☐

Theme: Discussing comorbidities at MDT meetings

7.1 Key points from policy and guidance

Cancer policy states that patients' demographic profiles and comorbidities should always be considered at MDT meetings. (11)

Guidelines for **Mental Health**, **Memory Clinic** and **Heart Failure** MDTs all emphasise the importance of identifying and managing comorbidities but there are no specific references to whether or how this information should be incorporated into MDT discussions. (23-26)

7.2 Key points from the research literature

99% of MDT members responding to an online survey agreed that patient comorbidities should always be considered when making a decision in the MDT meeting. (12)

Inadequate information on comorbidities has been associated with failure to make decisions in MDT meetings. (19, 27)

Having insufficient information on comorbidities has been reported to be an important cause of MDT decision non-implementation. (28)

7.3 Our research findings

Quantitative findings

Our study found no association between discussion of comorbidities and decision implementation.

Qualitative findings

There was diversity within and between specialties with respect to the extent to which comorbidities were discussed. No team discussed this information routinely.

Team members considered discussion of comorbidities to be valuable.

7.3.1 Arguments in favour of including comorbidities in MDT discussions

Discussion of comorbidities helps to determine suitable treatment options and informs differential diagnosis (e.g. cognitive impairment and depression).

Presentation of comorbidities encourages a holistic approach to treatment, for example, by highlighting the need to consider both physical and mental health issues, and issues relevant to patient engagement and adherence.

Patients wanted their comorbidities to be considered and included in treatment plans.

7.3.2 Arguments against including comorbidities in MDT discussions:

Information on comorbidities is not always available (e.g. when discussing patients for the first time), and not always accurate (e.g. as a result of incomplete assessment).

When comorbidities are discussed routinely this can add to the volume of potentially irrelevant information to be considered by the team, which can hamper decision-making.

Patients may be stigmatised or stereotyped because of their comorbidity (e.g. obesity).

It is not always possible to identify in advance of an MDT meeting, and therefore to have the information available on, comorbidities which might be relevant to decision making.

7.4 Questions

Comorbidities should be routinely discussed at MDT meetings	**Strongly Disagree ....Strongly Agree**<br>1 2 3 4 5 6 7 8 9	**Don't know**<br>☐

A patient should only be discussed at the MDT meeting when information on comorbidity is available	**Strongly Disagree ....Strongly Agree**<br>1 2 3 4 5 6 7 8 9	**Don't know**<br>☐

Approaches to incorporate information on comorbidities in the MDT meeting:

A designated MDT member should speak to the patient about comorbidities before the patient is discussed at an MDT meeting	**Strongly Disagree ....Strongly Agree**<br>1 2 3 4 5 6 7 8 9	**Don't know**<br>☐

**(a)** There should be a structured discussion on each patient with a section on comorbidities (e.g. using a checklist)	**Strongly Disagree ....Strongly Agree**<br>1 2 3 4 5 6 7 8 9	**Don't know**<br>☐

Patients' past medical history should routinely be available at the MDT meeting	1 2 3 4 5 6 7 8 9<br>**Strongly Disagree ....Strongly Agree**<br>1 2 3 4 5 6 7 8 9	☐<br>**Don't know**<br>☐

Do you have any other suggestions about how to incorporate comorbidities into MDT meeting discussions?

Theme: Discussing patients holistically

We use the term holistic to mean discussions which consider psychosocial in addition to biomedical issues.

8.1 Key points from policy and guidance

Guidance on effective **Cancer** MDT meetings states that patient psychosocial information should be collated and summarised before the MDT meeting wherever possible and presented where relevant. (29)

There is no specific MDT guidance on holistic discussion in **Mental Health**, **Memory** and **Heart Failure** MDT meetings. Nevertheless guidelines for all these disease types state that patient circumstances should be taking into account when managing their care. (2, 26, 30)

8.2 Key points from the research literature

Cancer research states that holistic MDT meeting discussions about patients lead to better care. (31)

An online survey of cancer MDT members found that 98% agreed that patients' psychosocial care issues should always be considered in the MDT meeting. (12)

A review of the evidence for Heart Failure MDTs argued that patient-centredness, and a concern for patient attitudes and experiences, is necessary to ensure high quality care. (32)

8.3 Our research findings

Qualitative findings

There was a distinction between teams which provided a sense of the individual as well as their condition or symptoms, and those teams where the emphasis was largely on the 'presenting complaint'.

In discussions and when making decisions, there was also a distinction between teams which relied predominantly on biomedical information and those that regularly discussed and included psychosocial information.

In teams where we observed frequent mention of psychosocial factors (mainly **Mental Health** and **Memory MDTs**), a small number of patients reported that they were not satisfied with their care and had negative opinions of MDT meetings.

Although in **Heart Failure** and **Cancer** teams the focus of discussions was predominantly on biomedical information, patients stated that they were satisfied with their care and had positive opinions of the MDT meeting.

8.3.1 Arguments in favour of routinely discussing patients holistically

Patients want to be recognised as individuals, not as ‘presenting complaints’.

Some patients worried that they might be misrepresented in meetings due to a lack of sufficient psychosocial information.

Some patients believed that MDT members without holistic knowledge of their case should not participate in the MDT discussions about them.

Most MDT members believed that when making decisions it is important to discuss psychosocial issues. However, even when this information was available, it was not always discussed.

Some MDT members believed that there is currently insufficient emphasis on psychosocial issues.

8.3.2 Arguments against routinely discussing patients holistically

MDT members believed that they included information on patients’ psychosocial circumstances *where relevant.*

Some MDT members believed it would not change the discussion/decision making.

Some MDTs discuss patients who are based at different hospitals so it is not always feasible to gather psychosocial information in time for the MDT meeting.

8.4 Questions

Statement	Strongly Disagree ....Strongly Agree	Don’t know
Relevant psychosocial issues for patients presented to each type of MDT should be identified and agreed by the MDT	1 2 3 4 5 6 7 8 9	☐

Statement	Strongly Disagree ....Strongly Agree	Don’t know
Case presentation should routinely include a brief introduction of the patient and relevant psychosocial characteristics, otherwise the case should not be discussed	1 2 3 4 5 6 7 8 9	☐

Statement	Strongly Disagree ....Strongly Agree	Don’t know
The MDT member who presents the case should routinely consider psychosocial factors and ensure that relevant information is available at the meeting	1 2 3 4 5 6 7 8 9	☐

Theme: Incorporating patient preferences about treatment into MDT discussions

9.1 Key points from policy and guidance

Guidance on effective MDT meetings in **Cancer** states that MDTs should take account of the patient's views, preferences and circumstances wherever possible. (29)

Guidelines for **Mental Health**, **Memory** and **Heart Failure** state that patient preferences should be taken into account when managing their care, although these do not relate specifically to the MDT meeting. (2, 26, 30)

9.2 Key points from the research literature:

An online survey of cancer MDT members found that 95% agreed that patient views/preferences should be presented at the MDT meeting by someone who has met the patient. (12)

Studies of cancer MDTs found that some treatment decisions made in MDT meetings were not implemented due to patient choice, and have suggested that this was due to a failure to consider patient preferences during the meeting. (28, 33)

A study of a single cancer MDT found that information about the patient's views, or choices regarding possible treatments, were not routinely included in discussions. MDT members reported that patients' views should be sought *after* the MDT meeting rather than before the meeting. (9)

Even physicians who are highly trained in communication skills are not good at predicting the decision making preferences of patients. (34)

Changes in disease status may change a patient's decision making preference over time. (34)

9.3 Our research findings

Quantitative findings

Our study found no association between discussion of patient preferences and decision implementation.

We collected data on 5847 decisions, of which 83 decisions (1.4%) were recorded as not implemented due to patient/carer/family choice.

Qualitative findings

There was variation in the frequency of discussion of patient preferences between and within teams.

There was variation between MDT members in whether they believed patient preferences were considered adequately in the meeting.

Certain professional groups (Clinical Nurse Specialists in **Cancer** and **Heart Failure** MDTs, care-coordinators in **Mental Health** MDTs) were perceived to be advocates for patients and more likely to know their preferences.

The extent to which patient preferences for treatment alternatives were considered differed across disease types. In **Mental Health** MDTs treatment options were often restricted due to resource issues (e.g. unavailability of psychologists). Discussions therefore focused on whether patients wanted to receive the service offered. In contrast, in **Cancer** MDTs when patient preferences were discussed, these focused on treatment alternatives. Patient preferences were most likely to be mentioned if they differed from the opinion of the MDT.

9.3.1 Arguments in favour of routinely discussing patient preferences about treatment

Most patients wanted their treatment preferences to be considered (including treatment type and location for receipt of treatment).

Some patients explicitly challenged/rejected clinical advice or treatment (i.e. chemotherapy).

Patients identified specific issues (e.g. travel plans, desire to stay in family home, dosages) that would affect their management preferences.

Knowing patient preferences can save time in decision making.

Some MDT members said that patient preferences were not adequately considered in the meetings.

9.3.2 Arguments against routinely discussing patient preferences about treatment

MDT members believed that patient preferences are important but the MDT meeting is not always the best place to discuss these. Some suggested that it is more important to have an informed discussion with the patient after the MDT meeting (during which the risks and benefits of different treatments are explored so these can be fed back to the patient).

Spurious discussions on treatment alternatives which are not available are time wasting.

It may not always be appropriate to allow patient preferences to influence decision making (e.g. compulsory admissions, preferences based on racist or homophobic beliefs).

Patient preferences change over time.

Patient preferences are not always known, for example, when a new patient is being presented or a patient has been added to the list at very short notice.

The patient is the final decision maker and can discuss, accept or reject the decision when they discuss it following the MDT meeting.

Different patients want different levels of choice which depends, among other things, on the severity of illness e.g. for critical decisions, survival was paramount and patients did not always think that treatment preferences needed to be discussed.

Some patients believed that the MDT is a forum for reaching the best clinical decision and should not be limited by their preferences.

Patients acknowledged that the team can't know all factors that influence personal choice in advance.

Some patients believed that making decisions within the context of resource constraints is more important than taking account of patients' treatment preferences.

Some patients believed that the doctor was the expert and that it would be 'foolish' not to follow the advice they were given.

9.4 Questions

Statement	Strongly Disagree ....Strongly Agree	Don't know
Patients' treatment preferences should be routinely discussed at the MDT meeting and if not available the case should not be discussed	1 2 3 4 5 6 7 8 9	☐
Any MDT member who presents a case should discuss treatment preferences with the patient before the MDT meeting	1 2 3 4 5 6 7 8 9	☐
Patient preferences regarding available management options should be reported to the MDT meeting only if the clinician responsible for their care thinks it will alter the decision	1 2 3 4 5 6 7 8 9	☐
Patient preferences regarding available treatment options should be discussed with the patient after (rather than before) the MDT meeting	1 2 3 4 5 6 7 8 9	☐
Patients should be asked *before* the MDT how much they want to be involved in decision making about their treatment	1 2 3 4 5 6 7 8 9	☐
Patient preferences should not be routinely discussed, but when making a decision, the MDT should actively seek all possible treatment options, and discuss these with the patient after the meeting	1 2 3 4 5 6 7 8 9	☐

Theme: Patient awareness of MDT meetings

10.1 Key points from policy and guidance

Cancer policy states that patients should be given a written description of the MDT meeting at which they are discussed (6), and should be made aware of the purpose of the MDT, when it meets (29), its membership and the responsibilities of each team member. (13)

Cancer guidance also states that patients should be informed of the outcome of discussions within a locally agreed timeframe. (29)

Community Mental Health Team guidance states that patients and their family or carers should be provided with a description of the service, though it doesn't specify whether they should be given details about MDT meetings. (2)

Policy documents for **Memory** and **Heart Failure** do not specify whether patients should be made aware of the fact that they may be discussed at an MDT meeting.

10.2 Key points from the research literature

Most **Cancer** MDT members surveyed (96%) agreed that patients should be made aware that an MDT will be advising on their treatment/care. (12)

10.3 Our research findings

Qualitative findings

Cancer patients were more frequently aware that they were being discussed in MDT meetings than patients in other specialties, where patients were often unaware of the existence of an MDT meeting.

Although most patients did not object to being discussed by a team, a small number of **Mental Health** and **Memory Clinic** patients were uncomfortable about being discussed (due to confidentiality issues and a fear that they would be misrepresented).

10.3.1 Arguments in favour of raising patient awareness about MDT meetings

Many patients reported that they feel more confident when a decision is made by an MDT rather than one person.

Some patients said they want to know about the MDT meeting process and who is involved.

10.3.2 Arguments against raising patient awareness about MDT meetings

Some patients said they did not want this information.

Some patients explicitly stated that they did not want to be discussed in an MDT meeting. This could raise difficult issues when a clinician or key worker believes that discussion at the MDT meeting will result in a better treatment decision.

10.3.3 Arguments for giving patients the opportunity to opt out of being discussed by the MDT

Some patients did not want people they didn’t know discussing their care, and wanted their cases to remain confidential between them and their clinician.

Some patients didn’t feel they needed or wanted the services of the MDT.

Some patients were concerned that they may be misrepresented at the MDT meeting by their keyworker.

10.3.4 Arguments against giving patients the option of opting out of being discussed by the MDT

Most patients thought MDT discussions were useful and very few would opt-out, with the exception of some Mental Health patients.

Most MDT members reported that the MDT meetings added value to patient care and that it was the best approach available.

Benefits of MDT discussion would be lost (e.g. peer review of decisions).

10.4 Questions

Statement	Strongly Disagree ....Strongly Agree	Don’t know
All patients should be told if they are going to be discussed at an MDT meeting *before* the meeting otherwise they should not be discussed	1 2 3 4 5 6 7 8 9	☐
All patients should be explicitly given the choice of whether or not to be discussed at the MDT meeting	1 2 3 4 5 6 7 8 9	☐
Patients should not be given an explicit choice, but if they express concern about being discussed at the MDT meeting they should be allowed to opt out	1 2 3 4 5 6 7 8 9	☐

Theme: Patient attendance at MDT meetings

11.1 Key points from policy and guidance

Despite extensive guidance and policy across specialties recommending that service users and patients should be involved in their care decisions, there are no specific guidelines about whether or not patients should attend MDT meetings.

11.2 Key points from the research literature

There is debate as to whether patients should attend MDT meetings and a lack of consensus on how best to involve patients in their care. (35)

Findings from a survey suggest that **Cancer** MDT members think it is impractical for patients to attend MDT meetings. (12)

11.3 Our research findings

Qualitative findings

The option of inviting patients to attend the meeting was not discussed in any of the MDT meetings we studied.

Patients' views and experiences were fed into MDT discussions in an ad hoc rather than a systematic manner. For example, in **Cancer** and **Heart Failure** teams, nurses believed it was their role to represent the patient's views. In **Memory** and **Mental Health** teams, patients' perspectives and circumstances were frequently mentioned by whoever had recently seen the patient.

Occasionally, the person presenting the case had not met the patient, and so there was little information about the patient's perspective.

11.3.1 Arguments in favour of patients attending MDT meetings

A small number of patients we interviewed said that they would like to attend the meetings.

Some MDT members suggested that patients attending could provide immediate and accurate patient input.

11.3.2 Arguments against patients attending the MDT meetings

A number of MDT members and patients believed that clinical decisions should *initially* be independent of patient input.

Practical barriers to patients attending MDT meetings include room size, suitability for accommodating sick patients and giving patients adequate advance notice of the time of the MDT meeting.

Patient attendance would change the content of discussions (e.g. use of humour, criticising other services, ethical dilemmas).

The meeting would need to be longer to accommodate patient attendance.

Some patients stated that attending the meeting might be upsetting and some discussions would be difficult for them to understand.

Most patients did not want to attend MDT meetings, but suggested alternative ways of feeding into the MDT discussion.

In some MDT meetings, patients are discussed before they meet the clinician or key worker in charge of their treatment and therefore attendance is not feasible.

In urgent cases there might be no time to arrange for patients to attend.

11.4 Questions

Patients should be given the option of attending MDT meetings	**Strongly Disagree ....Strongly Agree**<br>1 2 3 4 5 6 7 8 9	**Don't know**<br>☐

A number of alternative approaches to incorporating patient views and preferences into MDT decision making were suggested. Please rate the following:

Patients should not be presented at the MDT meeting unless there is someone present who has met with them at least once before the meeting, even if this postpones discussion of that patient	**Strongly Disagree ....Strongly Agree**<br>1 2 3 4 5 6 7 8 9	**Don't know**<br>☐

Patients should be given the opportunity to provide information in advance of the MDT meeting to ensure the information presented is accurate and comprehensive	**Strongly Disagree ....Strongly Agree**<br>1 2 3 4 5 6 7 8 9	**Don't know**<br>☐

Patients could provide information in the following ways. Please rate:

Patients should be able to provide information by having direct access and the ability to modify their medical records	**Strongly Disagree ....Strongly Agree**<br>1 2 3 4 5 6 7 8 9	**Don't know**<br>☐

Patients should be given the option to provide a written summary for the meeting	**Strongly Disagree ....Strongly Agree**<br>1 2 3 4 5 6 7 8 9	**Don't know**<br>☐

Patients should be given the option to provide audio recorded input to the meeting	**Strongly Disagree ....Strongly Agree**	**Don't know**

	1	2	3	4	5	6	7	8	9	☐

Theme: Providing feedback to patients on the outcome of MDT discussions

12.1 Key points from policy and guidance

There is extensive policy and guidance on the need to ensure that all patients are given appropriate information regarding their condition and treatment. (2, 30, 36)

Cancer policy states that individual clinicians will be responsible for discussing the MDT's recommendations with their patients, who should have the opportunity to be informed of the outcome of MDT meetings. (13)

There is no similar guidance for **Heart Failure**, **Memory** or **Mental Health** MDTs.

12.2 Key points from the research literature

A survey of cancer patients' views demonstrated that patients want to know details of their condition, treatment and side effects. (37)

A large **Cancer** study found that the vast majority of cancer patients want a great deal of specific information concerning their illness and treatment. (38)

Clinicians tend to underestimate the amount of information that patients require. (39, 40)

Whilst patients want basic information on diagnosis and treatment, not all want further information at all stages of their illness. (41)

12.3 Our research findings

Qualitative findings

Feedback to patients following an MDT meeting differed across teams, with some routinely discussing the MDT outcome with patients, while in others, patients were not necessarily told the outcome of the MDT discussion.

Patients' experiences of feedback varied, and different patients wanted different kinds of information fed back to them.

12.3.1 Arguments in favour of giving patients choices about the content and format of feedback from MDT meeting discussions

Patients want different levels of feedback on important information.

Some patients wanted to know about all treatment options discussed, including those that were ruled out by the team.

Patients' preferences for the mode of feedback varies (e.g. verbally, in writing).

12.3.2 Arguments against giving patients choices about the content and format of feedback from MDT meeting discussions

MDT members said that the team use professional judgement to decide the level and content of feedback appropriate for each patient at a given time (e.g. according to patient anxiety levels and comprehension).

Most patients don't want to know all aspects of care management.

Some patients stated that specialised information could be confusing for patients and patients can ask for further information if they wish to know more.

Some patients said that additional feedback would not change their management.

12.4 Questions

Statement	Strongly Disagree ....Strongly Agree	Don't know
Patients should be given feedback on which professional groups were present when they were discussed at the MDT meeting	1 2 3 4 5 6 7 8 9	☐
Patients should be given feedback every time they are discussed at an MDT meeting	1 2 3 4 5 6 7 8 9	☐
Patients should be given MDT meeting feedback only when decisions are made about their care	1 2 3 4 5 6 7 8 9	☐
Patients should be given feedback on all treatment options, even those rejected by the MDT	1 2 3 4 5 6 7 8 9	☐
Patients should be given written feedback about the outcome of the MDT meeting	1 2 3 4 5 6 7 8 9	☐
Patients should be given verbal feedback about the outcome of the MDT meeting	1 2 3 4 5 6 7 8 9	☐

Statement	Strongly Disagree ....Strongly Agree	Don't know
Patients should be able to choose the mode of MDT		

☐

meeting feedback (e.g. written, phone call, in clinic)	1 2 3 4 5 6 7 8 9	

Where it would be potentially inappropriate to share the content of an MDT discussion with the patient (e.g. where it may lead to unnecessary anxiety or disengagement from services), the decision not to feedback should be formally agreed and noted at the meeting by the team	**Strongly Disagree ……………………………Strongly Agree**<br>1 2 3 4 5 6 7 8 9	**Don't know**<br>☐

Theme: The role of research and evidence in MDT meetings

13.1 Key points from policy and guidance

Research is an essential component of modern **Cancer** care and MDTs should be aware of all current research for the cancers with which they deal. (1)

There is no guidance on the specific role of research in **Heart Failure**, **Memory** and **Mental Health** MDT meetings.

13.2 Key points from the research literature

Research has identified barriers to routinely incorporating the results of research into clinical decisions. These barriers include a lack of organizational support (e.g. restricted local access to information and unsupportive colleagues) as well as problems in interpreting research. (42)

13.3 Our research findings

Qualitative findings

There was variation across teams with respect to the frequency with which reference was made to research, with **Cancer** and **Heart Failure** teams explicitly drawing on research and evidence when making treatment decisions most often, followed by **Memory**, and then **Mental Health** MDT meetings where research and evidence were rarely mentioned.

Some teams used the MDT meeting as a forum to discuss research more generally, and not in relation to a specific patient or treatment decision.

13.3.1 Arguments in favour of discussing research evidence in MDT meetings

It can ensure that patients get the best treatment available, particularly in complex cases or where standard treatment options have not worked.

Evidence can be used as a means of resolving disagreements.

Discussions about research and evidence are a useful way of sharing knowledge and supporting learning.

For non-medical team members, using research evidence was seen as a way of capturing the attention of the rest of the team when they perceived that they weren't being listened to.

13.3.2 Barriers to discussing research evidence in MDT meetings

Discussion about research evidence can evolve into long arguments that are unrelated to the case being discussed.

There were different views amongst members of the team as to the role of research evidence. It was believed by some to comprise one component of holistic care, rather than the most dominant factor in decision making.

Research evidence is often inconclusive, and disagreements often arose when the evidence for different treatments was equivocal.

13.4 Questions

Statement	Strongly Disagree ....Strongly Agree	Don't know
There should be time within MDT meetings to discuss current and emerging research and evidence only in relation to the case discussed	1 2 3 4 5 6 7 8 9	☐

Statement	Strongly Disagree ....Strongly Agree	Don't know
There should be time within MDT meetings to discuss current and emerging research and evidence which is not specifically related to an individual case	1 2 3 4 5 6 7 8 9	☐

Statement	Strongly Disagree ....Strongly Agree	Don't know
Teams should be explicit about the research evidence that they are drawing on when making a decision in the MDT meeting	1 2 3 4 5 6 7 8 9	☐

Theme: Teaching as a function of MDT meetings

14.1 Key points from policy and guidance

Cancer policy and guidance states that MDT meetings provide an important educational opportunity, both for trainees and other team members. (1, 13, 29)

There is no explicit guidance on this issue for **Heart Failure**, **Mental Health** or **Memory** MDT meetings.

14.2 Key points from the research literature

Recommendations from the research literature include an allocation of time at the end of the MDT meeting for debriefing, discussion of educational points, and for the opinions of medical students and trainees to be heard. However, it is also acknowledged that '*these frenetic business meetings…often run beyond their allocated time slots'.* (43)

A national survey of cancer MDT members showed that MDT meetings have an important role in sharing learning and best practice with peers. (12)

14.3 Our research findings

Qualitative findings

There is evidence of teaching in some teams, but not all.

Examples of teaching include junior doctors presenting patients to the MDT meeting and being questioned about specific drugs, or individuals sharing information from their particular specialty as a form of interdisciplinary education. There were also examples of team members being given advice on how to present cases to the team.

14.3.1 Arguments in favour of teaching in the MDT meeting

Learning from members of the team, particularly from different professions or specialties, was perceived to be a valuable part of the meeting.

The MDT is a unique opportunity for multidisciplinary education for students, junior members of staff and for professionals.

Teaching makes the meetings more inclusive and more stimulating.

14.3.2 Arguments against teaching in the MDT meeting

Concerns were raised about the practicalities of teaching in MDT meetings, particularly given time constraints.

Some MDT members reported that students were not always able to keep up with the pressurised pace of the meeting.

Some MDT members reported that in reality there was little scope for professional development in MDT meetings.

14.4 Questions

Teaching should be a function of MDT meetings provided it does not add to the length of meetings	**Strongly Disagree** ....**Strongly Agree**<br>1 2 3 4 5 6 7 8 9	**Don't know**<br>☐

Teaching should be a function of MDT meetings even if it means meetings will be longer	**Strongly Disagree** ....**Strongly Agree**<br>1 2 3 4 5 6 7 8 9	**Don't know**<br>☐

Do you have any suggestions about how teaching can be incorporated into MDT meetings?

Theme: Recruitment to trials in MDT meetings

15.1 Key points from policy and guidance

According to national **Cancer** policy, a member of the core MDT should be nominated as the person responsible for ensuring that recruitment into clinical trials and other well designed studies is integrated into the function of the MDT. (16)

MDT meetings have an important role to play in **Cancer** research, particularly in increasing recruitment into clinical trials (1) and discussing clinical trials and audit results. (13)

Memory Services for early diagnosis and intervention services should provide opportunities for people with dementia to be included in research studies, although there is no explicit reference to the MDT meeting as a mechanism for doing so. (7)

This is also the case for **Heart Failure** and **Mental Health** where guidance on MDT meetings is much less detailed.

15.2 Key points from the research literature

Research has shown that recommendations to recruit to trials made in cancer MDT meetings significantly increases trial screening rates and may improve recruitment. (44)

15.3 Our research findings

Qualitative findings

There was wide variation between MDT meetings in how frequently clinical or research trials were discussed.

Specific mechanisms for recruitment to clinical trials included:

- having a dedicated research nurse or clinical trials practitioner at the MDT meeting, although these individuals did not generally contribute to discussions
- reference on an ad hoc basis to eligibility of a specific patient for a clinical or research trial by a member of the team (most commonly the Lead Consultant)
- team members or visitors giving a brief reminder to the team about specific trials they were involved with to raise awareness within the team.

15.3.1 Arguments in favour of discussing recruitment to trials in the MDT meeting

Creating a systematic approach to discussing trial recruitment in the MDT meeting could have a beneficial effect on recruitment and on referral awareness and activity.

A systematic approach would ensure that eligible patients are identified at an early stage to be recruited.

15.3.2 Arguments against discussing recruitment to trials in the MDT meeting

Becoming overly research focused may be at the expense of the individual patient under discussion, for example if there is a conflict between clinical needs and research needs.

There are differences in staffing and funding levels of research and activity across MDTs (e.g. some teams do not have dedicated research nurses or trials practitioners) so the mechanism for formally recruiting to trials in MDT meetings may not exist.

15.4 Questions

	**Strongly Disagree ....Strongly Agree**	**Don't know**
MDT meetings should be a forum for recruiting patients to clinical trials	1 2 3 4 5 6 7 8 9	☐

If you agree:

	**Strongly Disagree ....Strongly Agree**	**Don't know**
There should be a formal mechanism for discussing recruitment to trials in MDT meetings (for example, having clinical trials as an agenda item)	1 2 3 4 5 6 7 8 9	☐

	**Strongly Disagree ....Strongly Agree**	**Don't know**
All teams should have a designated person at each MDT meeting to help identify suitable patients for clinical trials		☐

	1	2	3	4	5	6	7	8	9	

Do you have any other suggestions about how to identify suitable patients for clinical trials in MDT meetings?

3 Theme: Monitoring the quality of MDT meetings

16.1 Key points from policy and guidance

Cancer policy states that the team should record both the identity of patients discussed at each MDT meeting and their treatment decisions. (6)

The National **Cancer** Peer Review programme involves both self-assessments and peer reviews which include auditing MDT meetings and reviewing MDT operational policies. (6)

Policy for **Heart Failure**, **Memory** and **Mental Health** MDTs does not specify whether or how the quality of MDT meetings should be monitored.

16.2 Key points from the research literature

A survey of Cancer professionals found that 86% agreed that MDTs need tools to support self-assessment/performance appraisal. 90% agreed that there should be agreed guidelines for how an MDT operates and how members work together. (12)

16.3 Our research findings

Quantitative findings

Mental Health MDTs scored consistently lowest on Team Climate Inventory items relating to quality monitoring (i.e. having clear criteria to meet in order to achieve excellence, monitoring each other to maintain high standards, and critically appraising weaknesses).

Qualitative findings

Quality of documentation varied widely across teams and specialties. In some teams action points were typed directly into patients' electronic records, in others actions were handwritten in a notebook. In the latter case patient names were often misspelled or omitted, making it impossible to link decisions and action points to particular patients. There was also variation in how easily accessible the minutes were to team members after the meeting. One team did not take any written notes or minutes of the meeting.

It was not possible to ascertain from medical records whether some decisions had been implemented due to a lack of documentation. This was most common in the **Mental Health** MDTs.

16.4 Questions

	Strongly Disagree ....Strongly Agree	Don't know
The objectives of the MDT meeting should be reviewed yearly	1 2 3 4 5 6 7 8 9	☐

Once a team has established a set of objectives for the meeting, the MDT should be audited against these goals (e.g. biannually)	**Strongly Disagree** ....**Strongly Agree**<br><br>1 2 3 4 5 6 7 8 9	**Don't know**<br><br>☐

All action points should be recorded electronically	**Strongly Disagree** ....**Strongly Agree**<br><br>1 2 3 4 5 6 7 8 9	**Don't know**<br><br>☐

Implementation of MDT decisions should be audited annually	**Strongly Disagree** ....**Strongly Agree**<br><br>1 2 3 4 5 6 7 8 9	**Don't know**<br><br>☐

Where an MDT meeting decision is changed, the reason for changing this should always be documented	**Strongly Disagree** ....**Strongly Agree**<br><br>1 2 3 4 5 6 7 8 9	**Don't know**<br><br>☐

There should be a named implementer documented with each decision	**Strongly Disagree** ....**Strongly Agree**<br><br>1 2 3 4 5 6 7 8 9	**Don't know**<br><br>☐

All MDTs should be audited through external peer-review	**Strongly Disagree** ....**Strongly Agree**<br><br>1 2 3 4 5 6 7 8 9	**Don't know**<br><br>☐

Thank you for taking the time to complete this survey. We look forward to discussing the results with you on the [date].

REFERENCES

1. Academy of Medical Royal Colleges. Intercollegiate Cancer Committee Educational Initiatives to Improve the Effectiveness of Cancer Multidisciplinary Teams. London: Academy of Medical Royal Colleges; 2009.

2. Department of Health. Mental Health Policy Implementation Guide: Community Mental Health Teams. London: Department of Health, March, 2001.

3. Royal College of Psychiatrists. Memory Services National Accreditation Programme: standards for memory services. London: Royal College of Psychiatrists, 2012.

4. National Institute for Health and Clinical Excellence. Services for people with chronic heart failure. National Institute for Health and Clinical Excellence, October, 2011.

5. West M, Alimo-Metcalfe B, Dawson J, El Ansari W, Glasby J, Hardy G, et al. Effectiveness of Multi-Professional Team Working (MPTW) in Mental Health Care. SDO 08/1819/215, 2012.

6. National Cancer Action Team. National Cancer Peer Review Programme Manual for Cancer Services: Gynaecology Measures. London: National Cancer Action Team, April, 2013.

7. Department of Health. Dementia Commissioning Pack: Service specification for dementia: memory service for early diagnosis and intervention. London: Department of Health, July, 2011.

8. Headrick L, Wilcock P, Batalden P. Interprofessional working and continuing medical education. BMJ. 1998;316(7133):771-4.

9. Kidger J, Murdoch J, Donovan J, Blazeby J. Clinical decision-making in a multidisciplinary gynaecological cancer team: a qualitative study. BJOG: An International Journal of Obstetrics & Gynaecology. 2009;116(4):511-7.

10. Borrill C, Carletta J, Carter A, Dawson J, Garrod S, Rees A, et al. The effectiveness of health care teams in the National Health Service: University of Aston; 2000.

11. National Cancer Action Team. The characteristics of an effective multidisciplinary team (MDT). London: National Cancer Action Team, February, 2010.

12. National Cancer Action Team. Multidisciplinary team members' views about MDT working: Results from a survey commissioned by the National Cancer Action Team. National Cancer Action Team, September, 2009.

13. National Institute for Clinical Excellence. Guidance on Cancer Services: Improving Outcomes in Haematological Cancers: The Manual. London: National Institute for Clinical Excellence, October, 2003.

14. Jalil R, Lamb B, Russ S, Sevdalis N, Green J. The cancer multi-disciplinary team from the co-ordinators' perspective: results from a national survey in the UK. BMC Health Services Research. 2012;12(1):457.

15. Whelan J, Griffith C, Archer T. Breast cancer multi-disciplinary teams in England: much achieved but still more to be done. Breast. 2006;15(1):119-22.

16. National Cancer Action Team. National Cancer Peer Review Programme Manual for Cancer Services: Haemato-oncology Cancer Measures London: National Cancer Action Team, April, 2013.

17. National Cancer Action Team. National Cancer Peer Review Programme Manual for Cancer Services: Skin Measures London: National Cancer Action Team, April, 2013.

18. Department of Health. Responsibility and accountability: moving on for new ways of working to a creative, capable workforce best practice guidance. London: Department of Health, February, 2010.

19. Lamb B, Sevdalis N, Benn J, Vincent C, Green J. Multidisciplinary Cancer Team Meeting Structure and Treatment Decisions: A Prospective Correlational Study. Annals of Surgical Oncology. 2013;20(3):715-22.

20. The Royal College of Radiologists. Cancer Multidisciplinary Team Meetings - Standards for Clinical Radiologists. London: Royal College of Radiologists, 2005.

21. Nouraei S, Philpott J, Nouraei S, Maude D, Sandhu G, Sandison A, et al. Reducing referral-to-treatment waiting times in cancer patients using a multidisciplinary database. Annals of the Royal College of Surgeons of England. 2007;89(2):113-7.

22. Lamb B, Sevdalis N, Mostafid H, Vincent C, Green J. Quality Improvement in Multidisciplinary Cancer Teams: An Investigation of Teamwork and Clinical Decision-Making and Cross-Validation of Assessments. Annals of Surgical Oncology. 2011(18):3535-43.

23. Department of Health. Choosing Health: supporting the physical needs of people with severe mental illness - commissioning framework. London: Department of Health, August, 2006.

24. Department of Health. Living well with dementia: a national dementia strategy London: Department of Health, February, 2009.

25. National Collaborating Centre for Mental Health. A NICE-SCIE Guideline on supporting people with dementia and their carers in health and social care. Guideline no 42. London: National Collaborating Centre for Mental Health, 2007 Contract No.: Guideline Number 42.

26. NHS Improvement. A guide for review and improvement of hospital based heart failure services. Leicester: NHS Improvement, November, 2011.

27. Stalfors J, Lundberg C, Westin T. Quality assessment of a multidisciplinary tumour meeting for patients with head and neck cancer. Acta Oto-laryngologica. 2007;127(1):82-7.

28. Blazeby J, Wilson L, Metcalfe C, Nicklin J, English R, Donovan J. Analysis of clinical decision-making in multi-disciplinary cancer teams. Ann Oncol. 2006;17(3):457-60.

29. Team NCA. The Characteristics of an Effective Multidisciplinary Team. 2010.

30. National Institute for Clinical Excellence. Dementia: Supporting people with dementia and their carers in health and social care. NICE Clinical Guideline 42. London: National Institute for Clinical Excellence, 2006.

31. Jefferies H, Chan K. Multidisciplinary team working: is it both holistic and effective? International Journal of Gynecological Cancer. 2004;14(2):210-1.

32. Sutherland K. Bridging the quality gap: Heart failure. London: The Health Foundation, 2010.

33. English R, Metcalfe C, Day J, Rayter Z, Blazeby J. A Prospective Analysis of Implementation of Multi-Disciplinary Team Decisions in Breast Cancer. The Breast Journal. 2012;18(5):459-63.

34. Bruera E, Sweeney C, Calder K, Palmer L, Benisch-Tolley S. Patient preferences versus physician perceptions of treatment decisions in cancer care. Journal of Clinical Oncology. 2001;19(11):2883-5.

35. Taylor C, Munro A, Glynne-Jones R, Griffith C, Trevatt P, Richards M, et al. Multidisciplinary team working in cancer: what is the evidence? BMJ. 2010;340:743-5.

36. National Institute for Clinical Excellence. Chronic heart failure: Management of chronic heart failure in adults in primary and secondary care. NICE Clinical Guideline no 108. 2010.

37. Meredith C, Symonds P, Webster L, Lamont D, Pyper E, Gillis C, et al. Information needs of cancer patients in West Scotland: cross sectional survey of patients' views. BMJ. 1996;313(7059):724-6.

38. Jenkins V, Fallowfield L, Saul J. Information needs of patients with cancer: results from a large study in UK cancer centres. British journal of cancer. 2001;84(1):48-51.

39. Fallowfield L, Ford S, Lewis S. No news is not good news: Information preferences of patients with cancer. Psycho-Oncology. 1995;4(3):197-202.

40. Degner L, L. K, D B, Sloan J, Carriere K, O'Neil J, et al. Information needs and decisional preferences in women with breast cancer. JAMA. 1997;277(18):1485-92.

41. Leydon G, Boulton M, Moynihan C, Jones A, Mossman J, Boudioni M, et al. Cancer patients' information needs and information seeking behaviour: in depth interview study. BMJ. 2000;320(7239):909-13.

42. McCaughan D, Thompson C, Cullum N, Sheldon T, Thompson D. Acute care nurses' perceptions of barriers to using research information in clinical decision-making. Journal of Advanced Nursing. 2002;39(1):46-60.

43. Sharma R, Shah K, Glatstein E. Multidisciplinary team meetings: what does the future hold for the flies raised in Wittgenstein's bottle? The Lancet Oncology. 2009;10(2):98-9.

44. McNair A, Choh C, Metcalfe C, Littlejohns D, Barham C, Hollowood A, et al. Maximising recruitment into randomised controlled trials: The role of multidisciplinary cancer teams. European Journal of Cancer. 2008;44(17):2623-6.
